# Middle Pleistocene Human Remains from Tourville-la-Rivière (Normandy, France) and Their Archaeological Context

**DOI:** 10.1371/journal.pone.0104111

**Published:** 2014-10-08

**Authors:** Jean-Philippe Faivre, Bruno Maureille, Priscilla Bayle, Isabelle Crevecoeur, Mathieu Duval, Rainer Grün, Céline Bemilli, Stéphanie Bonilauri, Sylvie Coutard, Maryelle Bessou, Nicole Limondin-Lozouet, Antoine Cottard, Thierry Deshayes, Aurélie Douillard, Xavier Henaff, Caroline Pautret-Homerville, Les Kinsley, Erik Trinkaus

**Affiliations:** 1 Unité Mixte de Recherche 5199, de la Préhistoire à l'Actuel: Culture, Environnement et Anthropologie (UMR 5199 - PACEA), Centre National de la Recherche Scientifique (CNRS), Université de Bordeaux, Talence, France; 2 Unité Mixte de Recherche 5199, de la Préhistoire à l'Actuel: Culture, Environnement et Anthropologie (UMR 5199 - PACEA), Centre National de la Recherche Scientifique (CNRS), Université de Bordeaux, Talence, France; 3 Unité Mixte de Recherche 5199, de la Préhistoire à l'Actuel: Culture, Environnement et Anthropologie (UMR 5199 - PACEA), Université de Bordeaux, Talence, France; 4 Unité Mixte de Recherche 5199, de la Préhistoire à l'Actuel: Culture, Environnement et Anthropologie (UMR 5199 - PACEA), Centre National de la Recherche Scientifique (CNRS), Université de Bordeaux, Talence, France; 5 Centro Nacional de Investigación sobre la Evolución Humana (CENIEH), Burgos, Spain; 6 Research School of Earth Sciences, The Australian National University, Canberra, Australia; 7 Institut national de recherches archéologiques préventives (INRAP) Grand Ouest, Centre archéologique de Grand Quevilly, Grand-Quevilly, France, and Unité Mixte de Recherche 7209 Archéozoologie, Archéobotanique, Muséum National d'Histoire Naturelle, Paris, France; 8 Unité Mixte de Recherche 7041, Archéologies et Sciences de l'Antiquité (UMR 7071 - ARSCAN), équipe Anthropologie des techniques, des espaces et des territoires au Pléistocène (ANTET), Maison René Ginouvès, Nanterre, France; 9 Institut national de recherches archéologiques préventives (INRAP) Nord-Picardie, Centre archéologique d'Amiens, Amiens, France, and UMR 8591 Laboratoire de Géographie Physique: Environnements Quaternaires et Actuels, Meudon, France; 10 Unité Mixte de Recherche 5199, de la Préhistoire à l'Actuel: Culture, Environnement et Anthropologie (UMR 5199 - PACEA), Université de Bordeaux, Talence, France; 11 Unité Mixte de Recherche 8591, Laboratoire de Géographie Physique: Environnements Quaternaires et Actuels, Centre National de la Recherche Scientifique (CNRS), Meudon, France; 12 Institut national de recherches archéologiques préventives (INRAP) Grand Ouest, Centre archéologique de Grand Quevilly, Grand-Quevilly, France; 13 Institut national de recherches archéologiques préventives (INRAP) Grand Ouest, Centre archéologique de Grand Quevilly, Grand-Quevilly, France; 14 Institut national de recherches archéologiques préventives (INRAP) Grand Ouest, Centre archéologique de Grand Quevilly, Grand-Quevilly, France; 15 Institut national de recherches archéologiques préventives (INRAP) Grand-Ouest, Centre archéologique de Carquefou, Carquefou, France; 16 Institut national de recherches archéologiques préventives (INRAP) Grand Ouest, Centre archéologique de Grand Quevilly, Grand-Quevilly, France; 17 Research School of Earth Sciences, The Australian National University, Canberra, Australia; 18 Department of Anthropology, Washington University, Saint Louis, Missouri, United States of America; University of Kansas, United States of America

## Abstract

Despite numerous sites of great antiquity having been excavated since the end of the 19th century, Middle Pleistocene human fossils are still extremely rare in northwestern Europe. Apart from the two partial crania from Biache-Saint-Vaast in northern France, all known human fossils from this period have been found from ten sites in either Germany or England. Here we report the discovery of three long bones from the same left upper limb discovered at the open-air site of Tourville-la-Rivière in the Seine Valley of northern France. New U-series and combined US-ESR dating on animal teeth produced an age range for the site of 183 to 236 ka. In combination with paleoecological indicators, they indicate an age toward the end of MIS 7. The human remains from Tourville-la-Rivière are attributable to the Neandertal lineage based on morphological and metric analyses. An abnormal crest on the left humerus represents a deltoid muscle enthesis. Micro- and or macro-traumas connected to repetitive movements similar to those documented for professional throwing athletes could be origin of abnormality.

## Introduction

**Figure 1:**
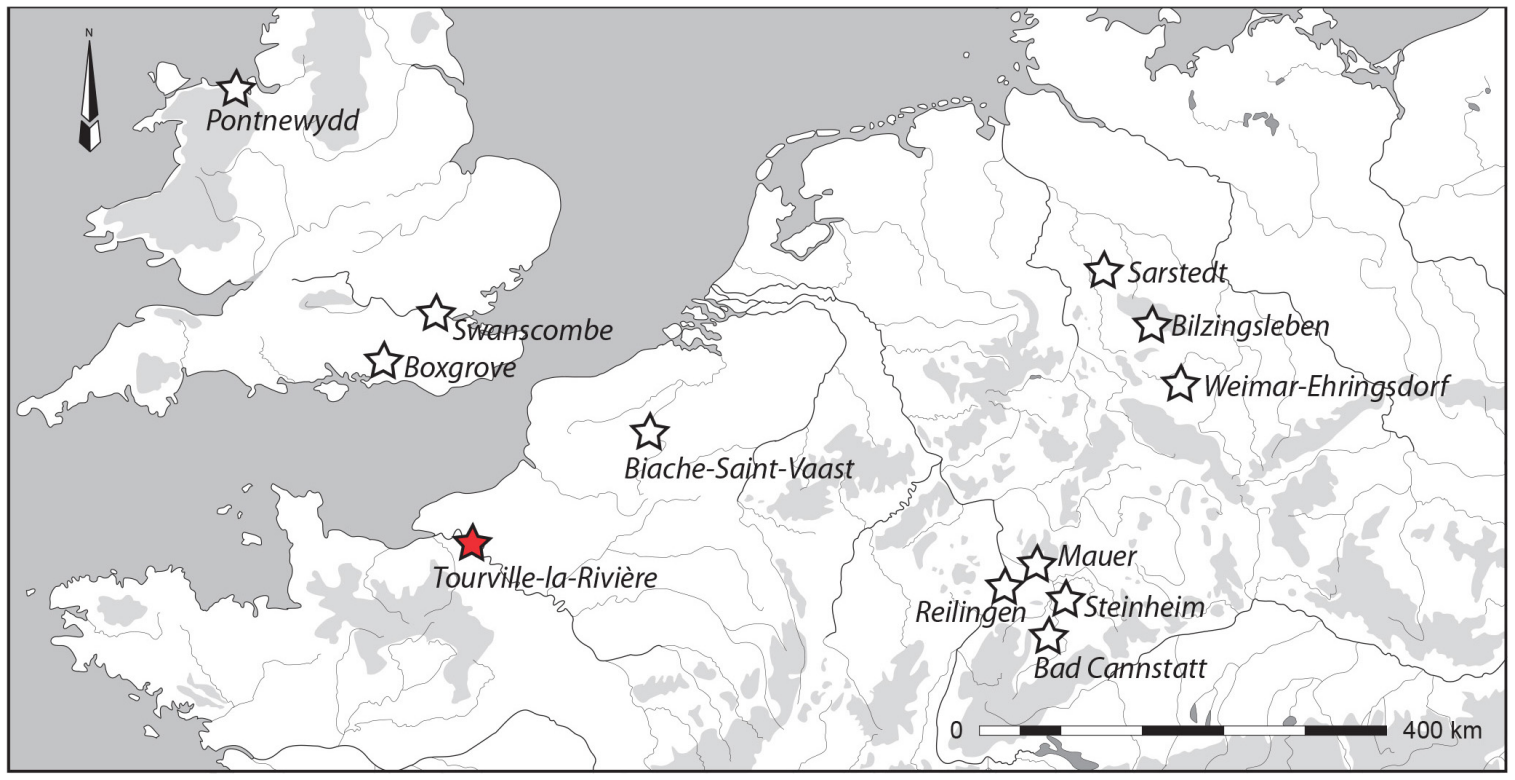
Location of the open-area site of Tourville-la-Rivière and other Northwest European (north to 45°N and west to 16°E) contexts, contemporaries of lower and middle Pleistocene (MIS-10-6), that have yielded human remains.

In Western Europe, Early and Middle Pleistocene sites that have produced human fossils generally reflect an earlier settlement of the Mediterranean region compared to northern Europe [1]–[11], despite the number of both recent and previous finds (Figure 1) coming from Germany [12]–[14] or England [15], [16]. Moreover, human fossils from the loessic plains or valleys of northern France remain extremely rare, limited to finds from Biache-Saint-Vaast [17]–[20]. Here, we report new human fossils from Tourville-la-Rivière (Seine-Maritime, France) that fill both geographic and chronological gaps in our understanding of this important period in European prehistory. Three left upper limb diaphyseal sections, most likely belonging to a single individual, were found in September 2010 during rescue excavations of this Middle Pleistocene site. This new find provides insight concerning the relationship of the Tourville remains to other Middle Pleistocene human fossils [12], [21]–[24]. We have applied U-series analyses on the human bones and combined US-ESR dating on faunal teeth to refine the chronology of Tourville-la-Rivière.

## Geography, Geomorphology, and Paleoenvironmental Context

**Figure 2:**
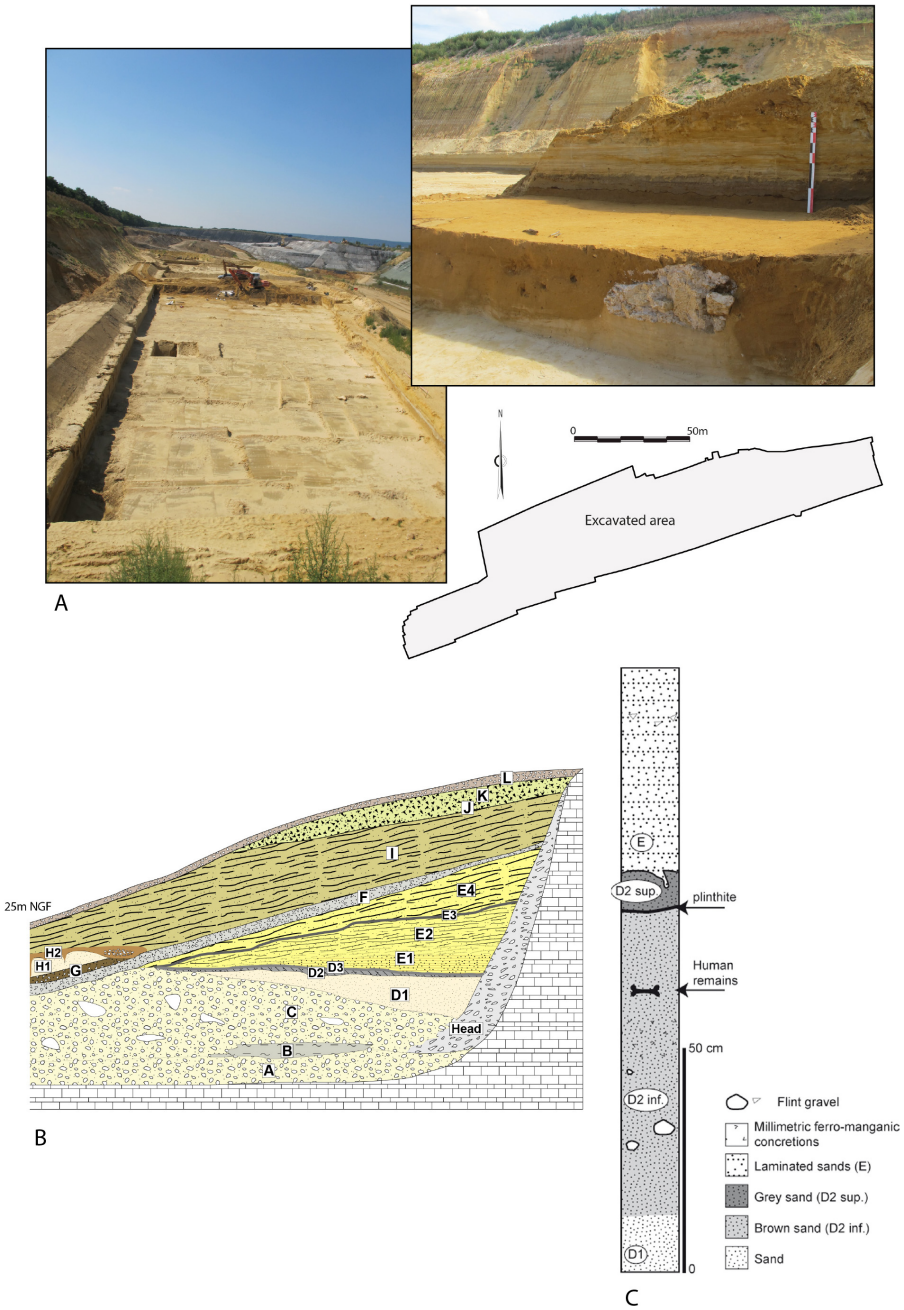
The Tourville-la-Rivière site. A: general view of the site during excavation; B: general stratigraphy of Tourville-la-Rivière (after [36], modified); C: stratigraphy of the excavated area.

The open-air site of Tourville-la-Rivière was discovered in 1967 in a Seine Valley gravel quarry (Figure 2A) that has been assiduously monitored by archaeologists, with several excavations having produced Early and Middle Palaeolithic faunal and lithic assemblages [25]–[33]. The site's substantial archaeological sequence lies on the lower terrace of the Seine River, abutting a chalky Cretaceous cliff (Figure 2B), which protected this >30 m thick geological formation.

This stratigraphic sequence, in the heart of a large meander of the Seine, comprises alluvial, estuarine, and continental sediments deposited from MIS 10 to 2 [34]–[38]. The majority of these deposits were lain down during the Saalian (MIS 8 to 6, or ∼300 ka to 130 ka) [39]–[41], making Tourville a reference sequence for Middle Pleistocene environmental change in northwestern Europe. The lowermost deposits are composed of coarse periglacial gravels and sands (layer C), overlain by fine-grained alluvial sediments (sands and silts), which are sub-divided into three layers (D1, D2 and D3). The upper-part of the sequence contains laminated sands (layer E) topped by periglacial deposits (layers F to K) composed of slope deposits and aeolian sands.

Based on malacological analyses [42], [43], the white sands comprising sedimentary sub-unit D1 accumulated during full interglacial conditions associated with the development of forest biomes. The top of D1 and D2 are dominated by snail species preferring more open habitats, suggesting a transition to a cold climatic period. Finally, D3 is characterised by species typical of cold and humid phases, clearly indicative of an Early Glacial phase.

## Archaeological Context and Human Behaviour of the Layer D2

Rescue excavations carried out by the Inrap (*Institut national de recherches archéologiques préventives*) in 2010 over approximately 2.5 acres focused on layer D2. Composed of a brown to grey hydromorphic soil developed on white alluvial sands (D1), the D2 deposits were divided into two sub-layers (D2 *sup* and D2 *inf*) by a goetithic plinthite, which can be followed laterally over several hundred meters. The human remains discussed here were recovered from layer D2 *inf* (Figure 2C).

Despite the extent of the excavated surface, very little archaeological material was recovered (1409 faunal elements and 726 lithics). The faunal material was documented from several different contexts (Figure S1 in File S1): scattered bones (predominantly herbivores), elements of isolated carcasses (limb portions, vertebral columns, skulls), or as part of a concentration composed of more than 600 pieces belonging to a dozen different herbivore, omnivore, or carnivore taxa. Both the D2 *inf* and *sup* faunal assemblages are dominated by herbivores and include a less substantial small mammal component (Figure S2 in File S1), reflecting a mix of wooded and non-arctic landscapes and a temperate interglacial climate. While malacological and pedo-sedimentary data from D2 *sup* are characteristic of the onset of a cooling phase (early MIS 6), the drop in temperatures that normally accompanies the emergence of a glacial phase is not systematically reflected in the faunal assemblages.

The role of the Seine River in the deposition and remobilisation of the Tourville fauna is unquestionable. Although it is clear that the faunal assemblage derives from multiple agents (natural processes, large carnivores, humans), it has been impossible to untangle their respective contributions. Nevertheless, bone splinters and green-stick fractures characteristic of marrow processing demonstrate the anthropic nature of the faunal material in certain excavation zones.

As with the faunal remains, the lithic artefacts are spread across the excavated area, separate from a small 9 m^2^ zone that represents a knapping concentration (Figure S3 in File S1). All of the raw material employed is local Senonian flint collected from the chalk cliff or local alluvium. The assemblage is composed of small pieces (chips, debris), core management flakes (cortical flakes or *éclats débordants*), rare non-Levallois cores, retouched tools (notches, becs, scrapers) or finished products, notably Levallois blanks and non-Levallois blades. The total absence of Levallois cores and the recovery of isolated Levallois products (elongated bi- and unipolar flakes) provide evidence for the substantial fragmentation of the reduction sequence and the importation of Levallois products to this zone [44] (Text S1 in File S1).

Several refit sequences demonstrate that cores and especially the largest unbroken products were transported away from the knapping zone (Figure S4 in File S1). Additional elements present in this zone can be connected to a slightly different Rocourt-type technology [45]–[49], which produced laminar flakes as well as blades (Text S2 and Figure S5 in File S1). Despite certain conceptual similarities with Levallois blade production, this Rocourt system exploits the core's center rather than surface. Although the objective is the production of elongated flakes and blades, this method also differs from Upper Palaeolithic-like Mousterian blade technology well-known during the Early Weichselian (MIS 5d-5a or 110 ka–70 ka) of northern Europe [49]–[55]. In this same region, Rocourt-type debitage is known from sites coeval with the beginning of the Weichselian (MIS 5d-5a) [56], [57]. The presence of this debitage method at Tourville thus provides yet another early example of this technology.

The small number of artefacts combined with the limited size of the single knapping concentration suggest rather ephemeral human occupations spread over a fairly substantial activity area [44], [58]. Elongated Levallois flakes and non-Levallois blades were likely designed for use in butchery or carcass processing, a probability reinforced by a preliminary functional analysis concerning a sample of these artefact types produced outside the excavated area [59] (Text S3 and Figure S6 in File S1).

## ESR and U-Series Dating

Five small pieces of human bone (Tourville A to E) were analysed for U-series isotopes along with a further eight equus or bovid teeth, five from D2 *inf* (TOUR1101, TOUR1102, TOUR1104, TOUR1105 and TOUR1108) and three from D2 *sup* (TOUR1103, TOUR1106 and TOUR1107) analysed by both U-series and ESR (Text S4, Figures S7.1, S7.2, Tables S1–S2 in File S1).

It was impossible to directly date the Tourville human remains, as each U-series analysis produced evidence for uranium leaching. Although eight animal teeth also indicate some U-uptake after burial, this is on a much smaller scale than the human bones, thus making them suitable for U-series dating. Our results indicate a minimum age of around 150 ka for layer D2 *inf* containing the human remains. Only three teeth could be used for combined U-series-ESR age calculations [60] due to leaching (Table 1), and they provide a weighted mean age of 194±14 ka. When a U-series-ESR model based on closed system [61] is applied, the obtained age is slightly older (211±15 ka). The best age range estimate (183 to 226 ka) is however that which takes into consideration the error envelopes of both models (open and closed systems) and thus accounts for all possible modes of continuous U-uptake (see File S1 for details of the dating procedure).

**Table 1:** ESR dating results calculated using the standard US [60] and the closed system (CS)-US [61] models.

Sample	US-ESR age (ka)	CS-US-ESR age (ka)
TOUR1101	184+26/−19	201±25
TOUR1102	174+17/−14*	188±21*
TOUR1103	208+28/−22	236±29
*Weighted mean*	*194±14/−11*	*211±15*

(*) For this sample, age calculation was performed using the dentine U-series data for the enamel as well. See File S1 for further details.

Our dating results combined with malacological data and current models of paleoenvironmental change [34], [35], [37], [38] indicate that layer C composed of coarse periglacial gravels can most likely be correlated with MIS 8, D1 with MIS 7, D2 with the transition from MIS 7 to MIS 6 and D3 with the onset of MIS 6.

## The Tourville Human Remains

This study involved the use of archaeological human remains recovered during salvage excavations, which were studied with the permission of the Inrap (France). All necessary permits or conventions were obtained for the described study, which complied with all relevant French regulations. The material studied consists of three upper limb bones labelled Tourville 2010 # 1174 (humerus), Tourville 2010 # 1175 (radius), Tourville 2010 # 1176 (ulna). The three bones are temporarily housed at the laboratory UMR-5199 PACEA (de la Préhistoire à l'actuel: culture, environnement, anthropologie) in Pessac, France.

### Discovery and taphonomy

**Figure 3:**
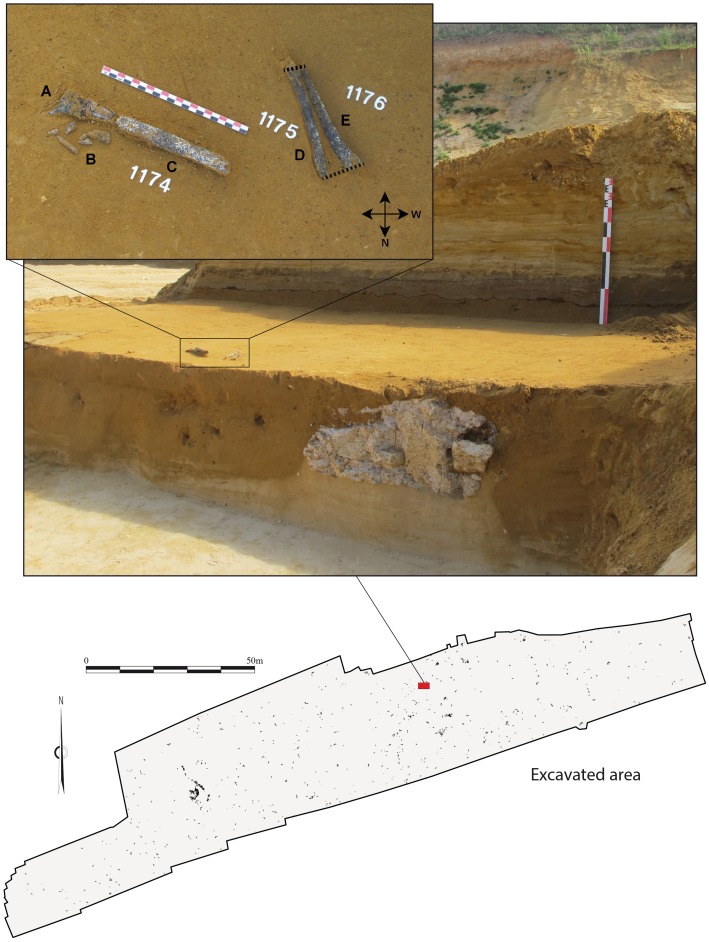
The Tourville human remains in situ. The posterior and medial surfaces were the first to be made visible for the radial (# 1175) and ulnar (# 1176) diaphyses, respectively, while the postero-medial surface of the humeral diaphysis (#1174) and posterior surface of the distal extremity were the first to be exposed. A: distal extremity of the humerus, posterior face; B: fragments of the distal portion of the humeral diaphysis. Several elements have since been refitted to the diaphysis (see Figure 4); C: the humeral diaphysis, medial to posteromedial face, proximal extremity to the north-west; D: radius, posterior face, proximal extremity to the north; E: ulna, medial face, proximal extremity to the north. Dotted lines indicate the alignment of the broken part of the distal and proximal extremities of the ulna and radius.

The Tourville human remains were discovered on September 10, 2010 (Figure 3) by A. Cottard and A. Thomann. Minor damage sustained during excavations and some taphonomic alterations are evident, and the postero-lateral portion of the lower third of the diaphysis is missing. The three shaft sections were oriented in approximately the same direction, which is common for elongated elements deposited in fluvial contexts or water lain deposits [62]–[64]. The combination of archaeothanatological inferences (the order in which the articulations dislocate) [65], the susceptibility of the bones to fluvial displacement, and an anatomical study (see below), suggest the most parsimonious scenario being the fluvial transport of the complete upper limb (with or without the hand), with subsequent minor post-depositional displacement and more dramatic damage affecting the arm and forearm. If the hand had been transported along with the three shaft fragments, the presence of faunal remains in anatomical position and their differential preservation (see above and Figure S1 in File S1) complicates an explanation for its absence.

**Figure 4:**
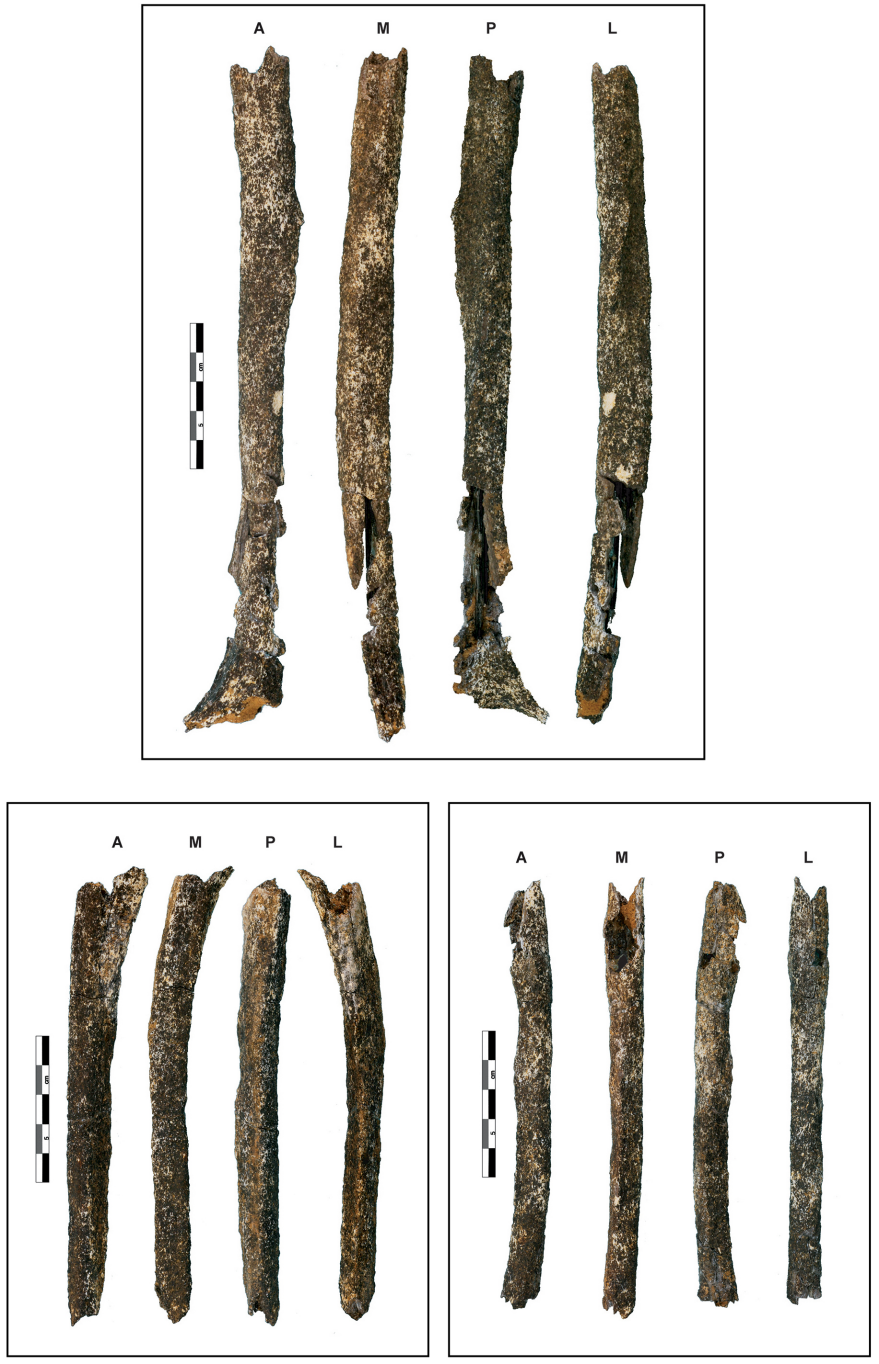
The Tourville left upper limb remains. Top: humerus; bottom left: ulna; bottom right: radius. For all the bones: A: anterior view; M: medial view; P: posterior view; L: lateral view.

The three incomplete bones belong to a left humerus, radius, and ulna that were partially crushed but have been restored and reconstructed (Figure 4, Text S5 in File S1). Their external cortical surface is heavily altered, stained dark grey to black and interspersed with small white patches, a coloration affecting the entire thickness of the cortical bone. This most likely results from depositional conditions tied to a hydromorphic sedimentary regime (standing water [62]–[64]). Small, rounded cracks are also visible, and their occasional star-like organisation may be a product of root etching [66]. Given their dimensions, these bones most likely belong to an adult or an older adolescent. Data concerning the comparative samples used in the morphometric analysis are described in File S1 (Text S6, Table S3 in File S1).

### The left humerus

**Figure 5:**
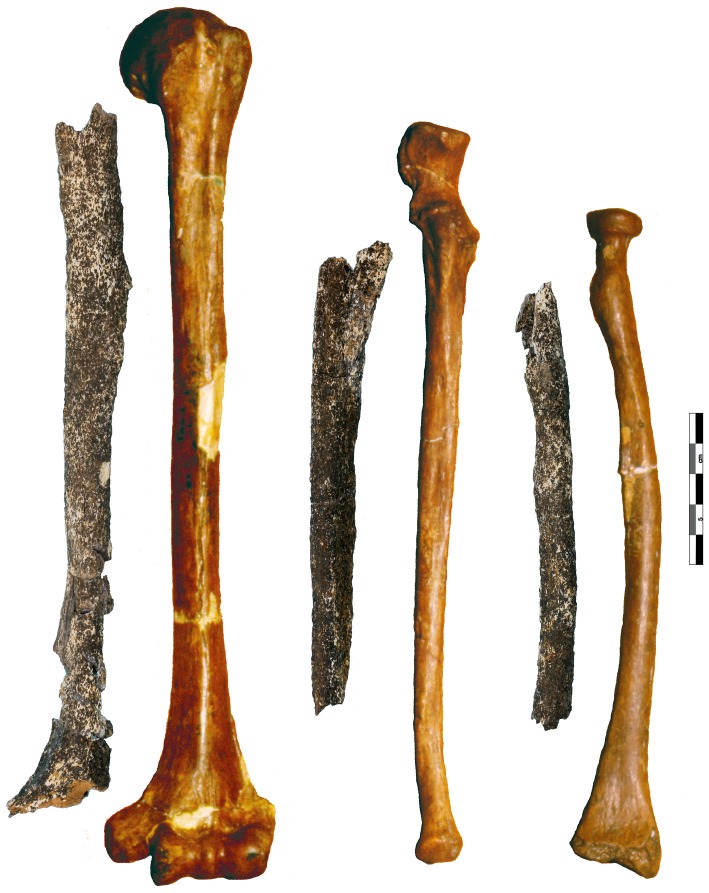
The Tourville 1 human remains in anterior view placed adjacent to the left arm bones of the Tabun 1 female Neandertal. The humeri are aligned according to the medial supracondylar crest, the ulnae using the brachialis tuberosity, and the radii using the radial tuberosity. Scale 5 cm.

The left humerus (Figure 4) consists of the eroded diaphysis from the region of the surgical neck proximally to the level of the olecranon fossa distally. It is not sufficiently intact to estimate its original length reliably. However, the preserved length (232 mm) is modestly longer than a similar length for the La Ferrassie 2 and Tabun 1 female Neandertal humeri (both with maximum lengths of 286 mm) (Figure 5), and close to the same dimensions for the male Feldhofer 1 and Regourdou 1 humeri (maximum lengths of 312 and 310 mm).

The position and form of the humeral deltoid tuberosity and a deltoid crest almost parallel to the lateral border are morphological traits more frequently reported in Neandertals than among modern humans [67], a pattern also documented in the Sima de los Huesos fossils [68], Tabun 1 [69], and the Feldhofer 1, La Chapelle-aux-Saints 1, La Ferrassie 1 and 2, and Regourdou 1 Neandertals [70]. The diaphyseal diameters and perimeters (Table S4 and Figure S8 in File S1) are situated in the lower part of the three comparative samples, confirming the modest diaphyseal dimensions of this bone. The midshaft diaphyseal index (see Table S4 in File S1) of the Tourville humerus is most similar to the pre-Neandertal sample (INDia: TOUR = 75.7; PNEAND = 75.7±4.4, n = 14) and reveals a transverse flattening at midshaft (platybrachy). On the other hand, an index considering one perimeter at the level of the deltoid tuberosity falls outside the 95% confidence interval for the two fossil groups, but within the variability of the modern human sample, a pattern due to an entheseal change affecting the posterior deltoid muscle insertion.

**Figure 6:**
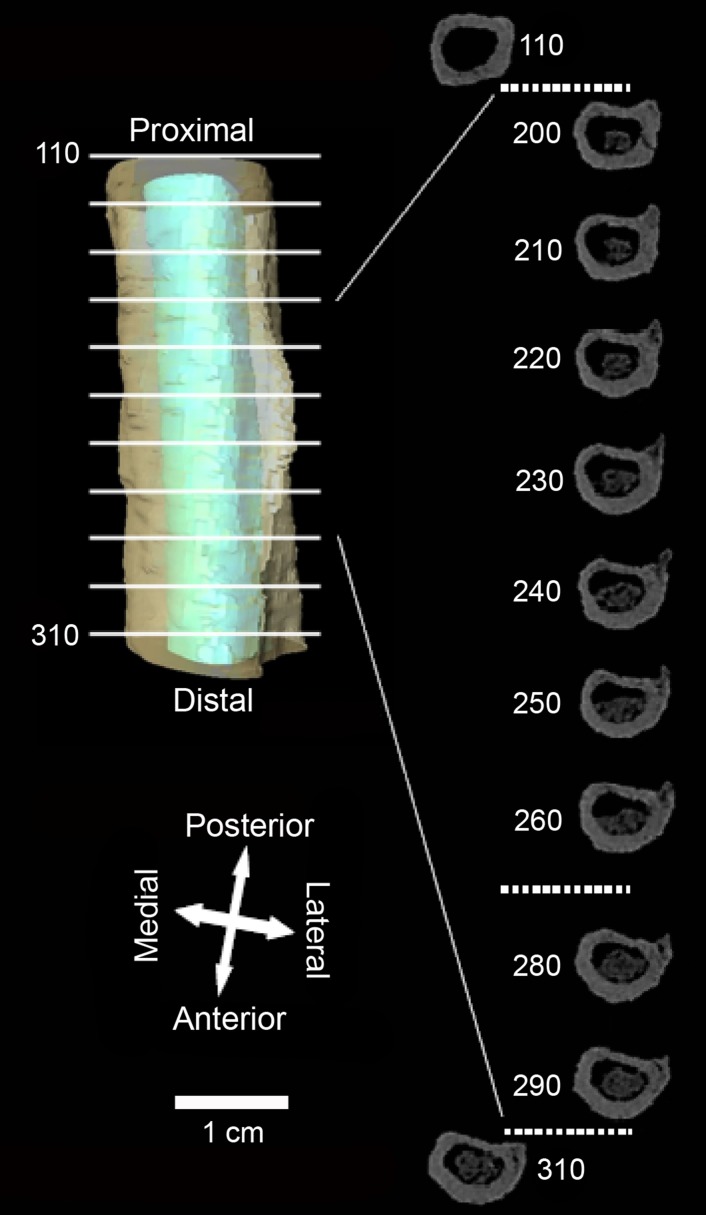
The enthesopathy modifying the posterior fasciculus of the deltoid muscle on the Tourville left humerus. Left: virtual reconstruction of the affected diaphyseal segment between horizontal cross-sections n° 110 and 310. In green: cortical bone, in blue: medullary cavity volume. Right: horizontal CT-scan of the same segment. Note both the substantial crest, which develops laterally and posteriorly, and the absence of any important relief at the area around insertion of the anterior muscle fasciculus.

A 4 cm long bony crest is evident on the Tourville humerus at the insertion site of the posterior deltoid muscle (Figure 4). A series of CT-scans (Figure 6) eliminates the possibility of taphonomic damage being responsible for this particular formation. The scans instead demonstrated the presence of an ‘entheseal change’, a recognizable feature on the surface of an enthesis [71]. The anterior view shows that the crest developed postero-laterally. The spur at the summit most likely represents an enthesophyte broken post-mortem. This bony projection tapers towards the proximal end of the humerus and is greater in length than in width.

In addition to the presence of the enthesophyte, the prominence of the crest falls outside the normal variability of Middle and Late Pleistocene European non-modern fossils. However, humerus III from La Sima de los Huesos [68] also has a well-developed crest at the posterior deltoid insertion (Figure S9 in File S1). While this type of crest has been reported for the deltoid insertion zone, its potential aetiologies vary. For example, although such formations seem to be more frequent among older individuals, they may also be connected to biomechanical factors (see references in [72]). Its development could therefore be linked with the habitual recruitment of the posterior deltoid muscle, which is implicated in the transverse extension/abduction of the arm and as a synergist to strong medial rotation of the arm [73], [74]. As such, the crest may be a result of micro- and or macro-traumas from repetitive movements, as in throwing sports requiring strong rotational stabilization of the shoulder [75].

The possibility of a biomechanical aetiology is further reinforced by the enthesophyte, which most likely results from a more sudden and violent trauma producing a tendinous or a bony avulsion [76]. Although tendon avulsions are most frequent when diaphyses are concerned, in some cases the bone tears away [77], the long-term consequences of which remain poorly documented. These may take the form of an osseous excrescence, at least in cases of fibrocartilaginous entheses [78], while in other instances damage can be minimal, sometimes even radiologically undetectable [79].

In order to assess whether the particular morphology of the specimen could be tied to constraints of habitual biomechanical loading, the cross-sectional geometric properties of the diaphysis were compared to those available for other Neandertal specimens [80], [81]. Midshaft and bicipital tubercle cross-sections at approximately 50% and 65% length, respectively, demonstrate a cortical bone of average thickness compared to the Late Pleistocene Neandertal sample (Table S5 in File S1). The second moment of inertia about the anteroposterior and mediolateral axes confirm the transverse flattening of the bone at midshaft, which is once again close to the Neandertal average (Table S5 in File S1). Finally, the polar moment of inertia (J), reflecting the bone's resistance to combined bending and torsional loading [82], is modest, but proper evaluation of it would require scaling to bone length and estimated body mass [83]. It would also necessitate comparisons only to humeri from the dominant or non-dominant arm (whichever one Tourville 1 represents), given the marked humeral diaphyseal asymmetry in most Late Pleistocene humans [84], [85]. Yet, since the Tourville 1 humeral length appears to have been above that of Tabun 1 (Figure 5), the only slightly greater J value suggests a more modest level of humeral diaphyseal hypertrophy.

**Figure 7:**
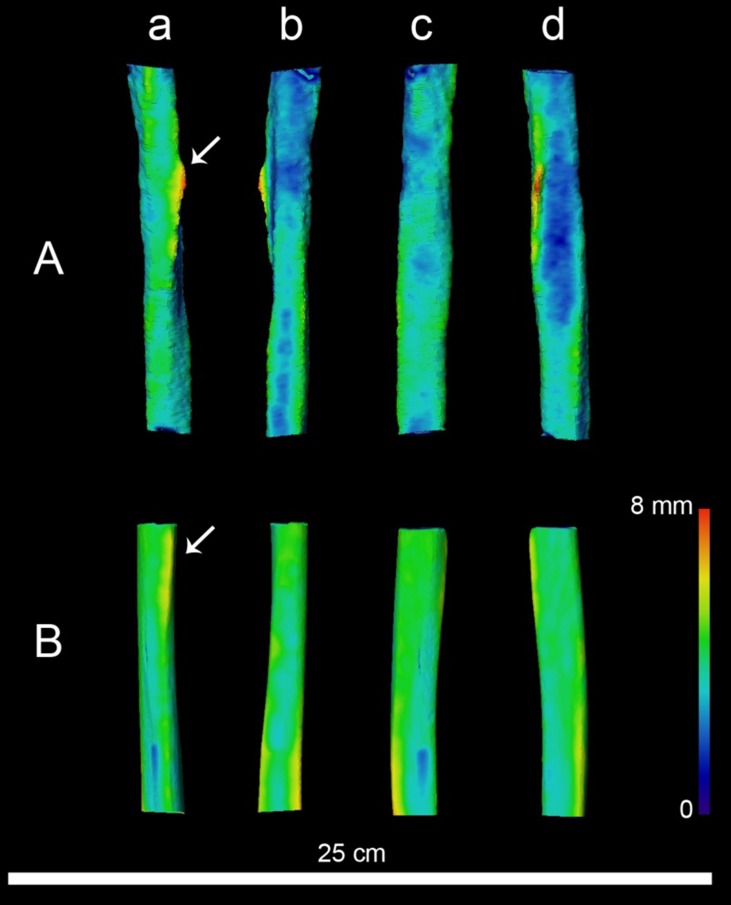
CT-based 3D mapping of the topographic distribution of the cortical bone at the proximal part of the humeral diaphysis (A) compared to the equivalent area (B, 6 cm below the deltoid tuberosity) on the humerus of the Krapina # 165 Pre-Neandertal (NESPOS data base, [**40**]) (B). Cortical thickness topographic variation was rendered using a chromatic scale increasing from dark blue (thin) to red (thick). The arrows indicate the position of the deltoid tuberosity in both shafts. a: anterior view, b: posterior view, c: medial view, d: lateral view.

CT-based 3D mapping of the topographic distribution of the cortical bone (Text S7 in File S1) rendered using a chromatic scale reveals important differences with one of the Krapina humeri available on NESPOS (Figure 7). A portion of these differences can be tied to taphonomic alterations as well as the poorly understood inter-individual variability within the Neandertal lineage. Moreover, the entheseal change is clearly visible in the topographic distribution of cortical bone as a zone of increased thickness.

### The left ulna

The left ulna (Figure 4) is an eroded diaphysis preserving the mid-supinator crest (proximally), as well as a partial crest on the anterolateral surface. The specimen is broken distally at approximately three quarters of its length which cannot be estimated. This apparently gracile ulna presents no abnormal relief and or developed muscular insertion. Similarities with Middle and Late Pleistocene Neandertal samples are evident, for instance, in the marked deviation of the proximal diaphysis compared to the bone axis [86]. The broad medial face is flat, as is also the case for La Ferrassie 1, and it is delimitated by clear and sharp crests compared to those of the lateral and anterior borders. The interosseous border is well marked and forms a pinched crest, which is a common feature of the Neandertals [87]. The medullary cavity narrows at its distal extremity due to the thickness of the cortical bone. The anteroposterior and transverse mid-diaphysis diameters (Table S6 and Figure S10 in File S1) are less than those of the reference sample, and the diaphyseal index is particularly low in comparison with the Neandertal sample.

### The left radius

The left radius (Figure 4) is an eroded diaphysis, which preserves the region of the neck in its proximal portion. Like the ulna, the specimen is gracile and broken distally at approximately three quarters of its length, which cannot be estimated. The medial surface of the diaphysis is missing above the radial tuberosity, as is most of muscle insertion zone. This radius shows no substantial relief or developed muscular insertions. Despite the radial tuberosity being eroded and represented only by its base, it is possible to determine that it is in a medial position relative to the interosseous border – an archaic *Homo* conformation more frequent among Neandertals (70.5%, n = 22) than early (8.8%, n = 40) and recent (2.8%, n = 496) modern humans [86], [88], [89]. A narrow, deep depression corresponding to the nutrient foramen is visible on the anterior surface near the interosseous border. The strongly marked interosseous crest is tear-drop shaped in radial section, and an interosseous tubercle is present between the radial tuberosity and the midshaft. Finally, the diaphysis presents a moderate lateral curvature compared to most Neandertals [70].

The Tourville radius is characterised by a thick cortical bone throughout the diaphysis (Table S7 in File S1). In terms of the perimeters and diameters at the level of the interosseous tubercle and midshaft, the Tourville specimen is more closely aligned with Middle Pleistocene mean and falls in the lower part of both the Late Pleistocene Neandertal and recent modern human ranges of variation (Figure S11 in File S1). However, the anteroposterior diameter is relatively high compared to the both the transverse diameter at midshaft and at the interosseous tubercle, producing a semicircular shaft section. The diaphyseal index at the interosseus tubercle also falls outside the range of variation for Late Pleistocene Neandertals characterised by flatter diaphyses.

## Taxonomic Attribution of the Tourville Human Fossil

Discussions regarding the Middle Pleistocene emergence of the Neandertals in Europe [12], [23], [90], [91] are primarily focused on cranial and dental autapomorphies, since few of their post-cranial features appear to be derived relative to earlier Pleistocene humans. This is particularly problematic for northern Europe, where the lack of comparative remains has limited the taxonomic attribution of post-cranial remains to being described simply as non-modern *Homo* (*e.g.* the Boxgrove tibia [16]). Although the Tourville human remains conform to the general Neandertal morphological pattern, they are insufficient by themselves to provide a secure taxonomic attribution. Yet, given the presence of uniquely derived Neandertal traits on the contemporaneous Biache-Saint-Vaast specimens [19], [92], it is therefore appropriate to place the Tourville fossil in the Neandertal lineage.

## Behavioural Interpretation

An unusual skeletal morphology, hitherto unknown for a Pleistocene fossil, is evident on the Tourville humerus, the abnormal bone formation at the deltoid tuberosity. An hypertrophied deltoid tuberosity is evident on a (probably Neandertal) right humerus from Khvalynsk [93] and on the left humerus of the Saint-Césaire 1 Neandertal [94]. Neither of these humeri, however, exhibits the kind of entheseal change evident on the Tourville humerus. Yet, at least one humerus (humerus III) from the Sima de los Huesos may have a similar crest.

Various causes can explain the crest on the Tourville humerus. Despite the multifactorial aetiology of entheseal changes in modern populations [95], we consider the simplest explanation for the altered muscular attachment to be biomechanical, with the enthesophyte at the summit of the humeral crest resulting from a single, more ‘violent’ trauma. The overall crest formation most likely results from repetitive micro- and or macro-traumas connected to the synergistic stabilization the arm associated with abduction and extension. Although the exact motion responsible for this entheseal change is difficult to determine, actions connected to throwing seem plausible [71], especially given the need for glenohumeral stability in spear throwing [96], as has been suggested for several Middle Palaeolithic contexts [cf.], [ 97]–[99].

Finally, there is a growing body of evidence for Middle [100]–[106] and Late [87], [106]–[115] Pleistocene non-modern human serious skeletal developmental variations, or minor ones, and associated survivorship. The Tourville humeral abnormality provides an additional case of a specific skeletal degeneration, which, in this case, is probably related to a specific activity or set of activities.

## Conclusion

Rescue excavations at the site of Tourville-La-Riviere produced substantial lithic and faunal material as well as a left humerus, ulna and radius belonging to the same individual and attributable to the Neandertal lineage. The site preserves a series of ephemeral but specialised MIS 7 occupations probably focused on butchery activities. The extensively excavated area (>2.5 acres) provides a window on a large part of the late Middle Pleistocene river valley, where humans transported stone tools between areas, discarding particular implements either where new ones were produced and then exported for later use or in locations where they were briefly used. This techno-economic data portrays a significant fragmentation of the reduction sequences [58] and a high mobility of the artefacts within the local environment of the Seine River valley.

While it is impossible to trace the taphonomic history of the human remains, their spatial organisation and anatomic proximity are similar to some of the faunal remains. In the absence of evidence for human or carnivore intervention, the most straightforward explanation for the presence of a human left arm at Tourville is its introduction to the site by fluvial transport. The morphological and metrical comparisons demonstrate this fossil to fall within the variability of the Neandertal lineage. Moreover, the Tourville humerus represents the first case of an unusual crest at the attachment site of the posterior deltoid muscle for a Pleistocene fossil.

Finally, the Tourville fossils are not only the oldest found in France during a rescue excavation, but also provide new material to what remains an extremely limited fossil sample from northwestern Europe, particularly in terms of post-cranial elements. Moreover, the trauma evident on the Tourville humerus may shed light on Neandertal behaviour. One possible explanation for the entheseal remodelling of the posterior deltoid muscle insertion is the habitual loading and torsional strain of the shoulder, possibly connected to repetitive movement. While interesting, this entheseal change probably had little bearing on the survival of the individual. The possible origins of this trauma may pose interesting questions about behavioural patterns among earlier Middle Palaeolithic humans.

## Supporting Information

File S1 **Headings and captions of the supporting text, supporting figures, and supporting tables. Text S1. Surface alteration of the lithic artefacts. Text S2. The Tourville example of non-Levallois laminar debitage. Text S3. Preliminary use-wear results. Text S4. U-series and ESR analyses. Text S5. Preservation of the Tourville fossils. Text S6. Comparison groups used in the morphometric analysis. Text S7. CT-scan methodology and results. Figure S1.Spatial distribution of the faunal remains. Figure S2. The D2** ***inf*** **faunal assemblage. Figure S3. Spatial distribution of lithic artefacts and focus on the knapping area. Figure S4. Refitting sequence comprising 46 pieces from the knapping concentration** (a). While mo**s**t elements of the reduction sequence are represented (waste, core management and shaping flakes, fragments of flakes and blades), several refitting sequences (b and c) show that the cores and largest products were exported. **Figure S5. Rocourt-type debitage.** 1- Elongated *éclats débordants* refit with laminar flake fragments. The negatives evince a bipolar debitage method producing either laminar flakes or blades. 2, 3 – Rocourt-type blades. **Figure S6. Examples of macro-wear (scarring) on Levallois products probably used to work soft animal materials. Figure S7.1. U-series results of five bone fragments of the human remains.** Top left: sample holder before analysis, left column: laser ablation analysis spots (the spot diameters are around 250 µm across); right column: U-series isotope results. When the ^230^Th/^238^U ratio is >^234^U/^238^U then leaching has occurred and no age can be calculated. **Figure S7.2. U-series results on eight faunal teeth.** Left: photos on the cross sections with laser ablation pits. The arrows indicate the analysis direction. Middle column: U-series isotope results. Right column: apparent U-series age estimates. Leaching is indicated by 400 ka age estimates, U-concentrations too low for age calculation are shown as zero ages. **Figure S8. Schematic representation of the adjusted Z-scores for Tourville humerus** relative to Pre-neandertal (blue curve), Neandertal (red curve), and extant modern human variability (green curve). Dmax = maximal diameter at mid-diaphysis; Dmin = minimal diameter at mid-diaphysis (M6); P6/12 = Perimeter at mid-diaphysis (M7a); P5/12 = Perimeter at the level of the deltoid tuberosity; INDTub = [(P5/12)/(P6/12)*100]; INDia = [(Dmax/Dmin)*100]. **Figure S9. Comparison of the deltoid lateral crest insertion** to (A) the left humerus of La Sima de los Huesos humerus III (anterior view) and (B) the crest (lateral view) from Carretero et al. [52]. Close-up (C) of the Tourville specimen (lateral view). Dotted line: orientation of the crest. **Figure S10. Schematic representation of the adjusted Z-scores of the Tourville ulna** relatively to the Neandertal variability (blue curve) and extant modern humans (green curve). Same legend as Table S4. **Figure S11. Schematic representation of the adjusted Z-scores of the Tourville radius** relatively to the Preneandertal variability (blue curve), Neandertal variability (red curve) and extant modern humans (green curve). Same legend as Table S5. **Table S1. U-series and ESR data obtained for all the Tourville samples. Table S2. Radioelement concentration obtained for the sediment. Table S3. Specimens used for comparing the Tourville upper limb dimensions and the cross-section properties of the humerus. Table S4. Dimensions of the Tourville humerus. Table S5. Cross-sectional geometric properties of the Tourville humerus and comparison with the Tabun C1 and Neandertal sample. Table S6. Dimensions of the Tourville ulna. Table S7. Dimensions of the Tourville radius.**
(ZIP)
