# Supplementary material for: Middle Pleistocene Human Remains from Tourville-la-Rivière (Normandy, France) and Their Archaeological Context
Source: PLoS One. 2014 Oct 8;9(10):e104111. doi: 10.1371/journal.pone.0104111 (PMC4189787; doi:10.1371/journal.pone.0104111)
Supplement: File S1 — Headings and captions of the supporting text, supporting figures, and supporting tables. Text S1. Surface alteration of the lithic artefacts. Text S2. The Tourville example of non-Levallois laminar debitage. Text S3. Preliminary use-wear results. Text S4. U-series and ESR analyses. Text S5. Preservation of the Tourville fossils. Text S6. Comparison groups used in the morphometric analysis. Text S7. CT-scan methodology and results. Figure S1.Spatial distribution of the faunal remains. Figure S2. The D2 inf faunal assemblage. Figure S3. Spatial distribution of lithic artefacts and focus on the knapping area. Figure S4. Refitting sequence comprising 46 pieces from the knapping concentration (a). While most elements of the reduction sequence are represented (waste, core management and shaping flakes, fragments of flakes and blades), several refitting sequences (b and c) show that the cores and largest products were exported. Figure S5. Rocourt-type debitage. 1- Elongated éclats débordants refit with laminar flake fragments. The negatives evince a bipolar debitage method producing either laminar flakes or blades. 2, 3 – Rocourt-type blades. Figure S6. Examples of macro-wear (scarring) on Levallois products probably used to work soft animal materials. Figure S7.1. U-series results of five bone fragments of the human remains. Top left: sample holder before analysis, left column: laser ablation analysis spots (the spot diameters are around 250 µm across); right column: U-series isotope results. When the 230Th/238U ratio is >234U/238U then leaching has occurred and no age can be calculated. Figure S7.2. U-series results on eight faunal teeth. Left: photos on the cross sections with laser ablation pits. The arrows indicate the analysis direction. Middle column: U-series isotope results. Right column: apparent U-series age estimates. Leaching is indicated by 400 ka age estimates, U-concentrations too low for age calculation are shown as zero ages. Figure S8. Schematic represen [file pone.0104111.s001.zip › File_S1.docx]

**Middle Pleistocene human remains from Tourville-la-Rivière (Normandy, France) and their archaeological context**

**Jean-Philippe Faivre^1^, Bruno Maureille^2^, Priscilla Bayle^3^, Isabelle Crevecoeur^4^, Mathieu Duval^5^, Rainer Grün^6^, Céline Bemilli^7^, Stéphanie Bonilauri^8^, Sylvie Coutard^9^, Maryelle Bessou^10^, Nicole Limondin-Lozouet^11^, Antoine Cottard^12^, Thierry Deshayes^13^, Aurélie Douillard^14^, Xavier Henaff^15^, Caroline Pautret-Homerville^16^, Les Kinsley^17^, Erik Trinkaus^18^**

^1^ UMR 5199 PACEA-PPP, CNRS, Université de Bordeaux, F-33405 Talence, France.

jp.faivre@pacea.u-bordeaux1.fr

^2^ UMR 5199 PACEA-A3P, CNRS, Université de Bordeaux, F-33405 Talence, France. b.maureille@pacea.u-bordeaux1.fr

^3^ UMR 5199 PACEA-A3P, Université de Bordeaux, F-33405 Talence, France. p.bayle@pacea.u-bordeaux1.fr

^4^ UMR 5199 PACEA-A3P, CNRS, Université de Bordeaux, F-33405 Talence, France. i.crevecoeur@pacea.u-bordeaux1.fr

^5^ Centro Nacional de Investigación sobre la Evolución Humana (CENIEH), Paseo de Atapuerca, 3, 09002-Burgos, Spain. mathieu.duval@cenieh.es

^6^ Research School of Earth Sciences, The Australian National University, Canberra ACT 0200, Australia. Rainer.Grun@anu.edu.au

^7^ INRAP Grand Ouest, Centre archéologique de Grand Quevilly, 76120 Grand-Quevilly, France. UMR 7209 Archéozoologie, Archéobotanique, Muséum National d’Histoire Naturelle, 75005 Paris, France. celine.bemilli@inrap.fr

^8^ UMR 7041 ArScAn-AnTET, Maison René Ginouvès, F-92023 Nanterre, France. stephanie.bonilauri@gmail.com
^9^ INRAP Nord-Picardie, Centre archéologique d’Amiens, 80000 Amiens, France. UMR 8591 Laboratoire de Géographie Physique : Environnements Quaternaires et Actuels, 92195 Meudon, France. sylvie.coutard@inrap.fr.

^10^ UMR 5199 PACEA-A3P, Université de Bordeaux, F-33405 Talence, France. m.bessou@pacea.u-bordeaux1.fr

^11^ UMR 8591 Laboratoire de Géographie Physique : Environnements Quaternaires et Actuels, CNRS, 92195 Meudon, France.

nicole.limondin@cnrs-bellevue.fr

^12^ INRAP Grand Ouest, Centre archéologique de Grand Quevilly, 76120 Grand-Quevilly, France. antoine.cottard@inrap.fr

^13^ INRAP Grand Ouest, Centre archéologique de Grand Quevilly, 76120 Grand-Quevilly, France. thierry.deshayes@inrap.fr

^14^ INRAP Grand Ouest, Centre archéologique de Grand Quevilly, 76120 Grand-Quevilly, France. aurélie.douillard@inrap.fr

^15^ INRAP Grand-Ouest, Centre archéologique de Carquefou, 44477 Carquefou, France. xavier.henaff@inrap.fr

^16^ INRAP Grand Ouest, Centre archéologique de Grand Quevilly, 76120 Grand-Quevilly, France. caroline*.*pautret*-*homerville*@*inrap*.fr*

^17^ Research School of Earth Sciences, The Australian National University, Canberra ACT 0200, Australia. leslie.kinsley@anu.edu.au.

^18^ Department of Anthropology, Washington University, Saint Louis MO 63130, USA. trinkaus@wustl.edu

**SUPPORTING INFORMATION**

**Supporting text**

**Text S1. Surface alteration of the lithic artefacts**

Given the alluvial context, the absence of a clear spatial association between different artefact categories may be indicative of fluvial displacement. However, although some isolated objects are patinated, their ridges and edges are undamaged. In order to confirm these initial observations, more detailed analysis of 65 lithic artefacts at middle magnification (7x - 45x) was carried out using a binocular microscope. Despite their white patina, 55 of 65 artefacts bear no traces of post-depositional alteration. The ridges and edges, modified or not, are remarkably well preserved; there is a near total absence of natural blunting or scarring/crushing and the active edges are extremely fresh and remain sharp.

Although ten pieces stand out given their rather well-developed lustre and rolled aspect, the absence of taphonomic alterations on the large majority of lithic artefacts confirm their excellent preservation, corroborating the fact that that they suffered only minimal post-depositional reworking. This, coupled with the excellent preservation of knapping area, tempers the role of water action in the distribution of the lithic artefacts. In our opinion, the nature and spatial distribution of the lithic material clearly indicates that the location was occupied by human groups.

**Text S2. The Tourville example of non-Levallois laminar debitage**

Rocourt debitage, named after the eponymous site of Rocourt in Belgium, is initiated from one of the core's narrowest edges and subsequently moves across the widest surface [1-2]. The recurrence of bipolar laminar removals insures that production can proceed with relatively little management of the surface's longitudinal convexities. The removal of elongated *éclats débordants* or cortically backed flakes with transverse negatives (crests in a broad sense) allow core reduction to pass from the exploitation of a surface to volumetric core conception (Figure S5)

**Text S3. Preliminary use-wear results**

A preliminary use-wear study using low and middle (7x to 45x) as well as higher magnification (100x and 200x) was carried out on a sample of 25 pieces. Macro- (scarring and edge blunting) and micro-traces (striations and polish) were observable despite the white patina (Figure S6). The location of the active edges, the materials worked, and motions are relatively varied and seem to correlate with the type of product (blades, flakes, presence or absence of retouch). However, despite the variable actions (longitudinal and transversal) and different worked material (animal and probably vegetal), the majority of objects were used longitudinally to cut soft (meat) and probably hard (tendons, cartilage, bone, etc.) materials. This preliminary use-wear study demonstrates that the analysed artefacts were used on-site, corroborating the hypothesis that resources (animal and mineral) available on the site were exploited by Neandertal groups.

**Text S4. U-series and ESR analyses**

**1. Introduction**

1.1. U-series analysis

With the advent of rapid U-series analysis using laser ablation ICP-MS, numerous human fossils have been analysed, some of which yielded significantly older ages than previously established [3-6]. U-series dating of bones is seriously compromised by the fact that bones can accumulate significant quantities of uranium following deposition. A range of models have been developed to account for this uranium uptake in order to provide a basis for open system dating. The diffusion-adsorption (D-A) model, developed by Millard [7] and Millard and Hedges [8], and refined by Pike [9] and Pike *et al* [10], is based on laboratory experiments and assumes a continuous diffusion of uranium from the exterior of a bone or tooth towards the interior, and that the partitioning between the bone and solution (groundwater) and U-concentration in the solution are constant. The bone is treated as a homogeneous medium. Under constant conditions, bone cross sections conforming to the D-A diffusion model are expected to have both u-shaped U-concentration and apparent U-series age profiles, with the apparent ages at the surface being closest to the correct age of the sample. Deviations from such ideal profiles can be explained either by leaching or changes in the solution's U-concentration. The D-A model was recently refined by Sambridge et al. [11], whose Diffusion-Adsorption-Decay (DAD) model expands the DA model for diffusion of ^234^U and its decay during the diffusion process. For a given volume in a bone, the DAD model postulates that ^234^U is continually resupplied by diffusion. As a result, ^234^U/^238^U ratios vary little over time, consequently DAD model ages are somewhat older than comparative DA results.

The problem in the analysis of teeth is that it is difficult to reconstruct the direction for U-diffusion, which may often be perpendicular to the cutting plane. Thus, for most teeth neither the DA nor the DAD model is applicable. Nevertheless, if U-leaching can be excluded, any closed system U-series result should be regarded as minimum age estimate. If leaching does occur, age assessment becomes difficult to impossible.

1.2. ESR analysis

The main problem of dating fossil teeth by ESR lies in the complexity of the dose rate for age calculation, which must include sample geometry, as well as all the components involved in the natural irradiation of the enamel layer (i.e., several dental tissues and sediment). Additionally, dental tissue is known to behave as an open system for uranium. Therefore, the modelling of uranium uptake into dental tissue over time is crucial for the dose rate assessment. Grün et al. [12] proposed to combine U-series and ESR data using the following function (US model): U(t) = U_m_ (t/T)^p+1^, where U(t) is the uranium concentration at time t, U_m_ is the measured, present-day uranium (U) concentration, T is the age of the sample and p is the uptake parameter.

For the US model, the U-uptake is assessed mathematically according to the present day U-series (U concentration, ^230^Th/^234^U and ^234^U/^238^U) measured in each tissue. As a consequence, only one combined US-ESR age fits the measured U-series and ESR dataset for a given sample. Depending on the position of the enamel used for dating, up to three U-series analyses have to be carried out (e.g. for horse teeth, in which the enamel sample may be intercalated between layers of cement and dentine). The US model describes only continuous uptake histories and the present-day U-concentration is considered the maximum value ever achieved. Consequently, U-leaching from dental tissue cannot be modeled [12], meaning that apparent U-series ages cannot be older than the EU-ESR ages, otherwise age calculations cannot be performed.

An alternative to the US-ESR is the CSUS ESR model that assumes that all uranium migrated into the skeletal tissue at the time given by the closed system U-series age [13]. For a given data set, the US-ESR model provides the minimum age estimate while the CSUS-ESR model a maximum age estimate. By using both models, the age ranges provided encompass all possible continuous U-uptake histories, as long as there are no leaching episodes.

**2 Samples**

Two sets of samples were analysed. The first set consisted of five small pieces of the human bone (Tourville A to E). These were analysed for U-series isotopes. A second set consisted of eight teeth, five were found in D2 *inf* (TOUR1101, TOUR1102, TOUR1104, TOUR1105 and TOUR1108) and three in D2 *sup* (TOUR1103, TOUR1106 and TOUR1107). These were analysed by both U-series and ESR. Four sediment samples were collected from Layer D2, three in direct contact with TOUR1104, TOUR1105 and TOUR1107. The dating process started at the end of 2011. As the original excavation finished in September 2010, *in situ* gamma dosimetry measurements were impossible. As such, four sediment samples were collected from Layer D2 and used for the calculation of the external gamma dose rate.

**3. Experimental**

3.1. U-series analysis

The U-series isotopes in dental tissues were analysed by laser ablation multi-collector ICP-MS at RSES (Canberra, Australia), using a custom-built laser sampling system interfaced between an ArF Excimer laser and a Finnigan Neptune (for details, see [14-15]. Data reduction followed established laser ablation ICP-MS protocols [16] using the dentine of a rhinoceros tooth from Hexian (sample 1118, see [17]) as a secondary matrix matched standard. At the time the Neptune mass spectrometer was equipped with an ion counter only, which necessitated the separate measurement of ^230^Th and ^234^U. Since the ^234^U/^238^U ratios usually change considerably less than the ^230^Th/^238^U ratios across a bone, only half as many ^234^U compared to ^230^Th measurements were carried out. Ages were calculated either using the nearest pair within a given dental tissue (e.g. TOUR1102) or the average ^234^U/^238^U ratio for a given dental tissue within a tooth (e.g. for section 2 of TOUR1101, see Figure S7.2).

3.2. ESR analysis

Fossil teeth were prepared according the CENIEH routine procedures (e.g. [18]): an enamel layer was mechanically separated from the other dental tissues and both inner and outer sides were slightly cleaned with a dentist’s drill to eliminate the external alpha particles contribution. Clean enamel fragments were then ground and sieved to recover the 100–200 µm size fraction. The powder was split into 10 aliquots and irradiated with a calibrated ^60^Co gamma source, using an exponential dose step distribution [19], with the following doses: 0, 50.3, 99.7, 250, 501, 1000, 1230, 1801, 3800 and 5000 Gy.

ESR measurements were carried out at CENIEH with a EMXmicro 6/1 Bruker ESR spectrometer equipped with a standard rectangular ER 4102ST cavity. Constant temperature measurement conditions at 18ºC were provided by a Thermo Scientific NESLAB ThermoFlex 3500 chiller. The temperature of the room is kept constant at 20 °C by an air conditioning unit. ESR measurements were performed at room temperature with the following acquisition parameters: 5-20 scans, 1 mW microwave power, 1024 points resolution, 15 mT sweep width, 100 kHz modulation frequency, 0.1 mT modulation amplitude, 20 ms conversion time and 5 ms time constant. The ESR intensities were extracted from T1-B2 peak-to-peak amplitudes of the ESR signal of enamel [20], normalized on receiver gain, number of scans and aliquot mass. All aliquots of a given sample were successively measured within a short time interval (<1 h). Fitting of the dose response curve was carried out with the Microcal OriginPro 8.5 software using a Levenberg-Marquardt algorithm by chi-square minimization. The data points were weighted by the inverse of the squared ESR intensity (1/I^2^) [21].

To check D_E_ reproducibility, measurements were repeated over several days without removing the enamel from the ESR tubes. The reproducibility of D_E_ estimations was better than 2.5%. The final D_E_ values were obtained by fitting a single saturating exponential (SSE) function [22] through the mean ESR intensities derived from the repeated measurements of each sample. The ESR data and relevant U-series results are shown in Table S1.

Combined US-ESR and CSUS-ESR ages were calculated with the DATA programme [23] using the following parameters: an alpha efficiency of 0.13±0.02 [24], Monte-Carlo beta attenuation factors from Marsh [25], dose-rate conversion factors from Guérin et al. [26], an estimated water content of 5±3 wt.% in dentine and cement and 20±10 wt.% in sediment. U, Th and K concentrations in sediment were determined by ICP-OES and ICP-MS analysis on samples collected in the vicinity of the fossil teeth (Table S2). The cosmic dose rate was calculated according to Prescott and Hutton [27-28]. The combined US-ESR and CSUS-ESR age estimates are shown in Table 1.

**4. Results and discussion**

The enamel sections for the ESR analyses were cut to have a sediment-enamel-dentine geometry where possible while the U-series was aimed at all dental tissues. Thus, several teeth have U-series results on cement while the ESR age calculations do not involve cement data where the enamel was not covered by cement. Also note that some isotopic ratios are different in the U-series section from the ESR calculations because only the interior of the enamel was used for the latter.

4.1. U-series analyses.

The U-series data on the human bone are shown in Figure S7.1. It can be seen that all ^230^Th/^238^U activity ratios are significantly higher than the ^234^U/^238^U ratios, indicating uranium leaching. This makes it impossible to calculate any numerical age estimates, or even to establish any minimum or maximum age estimates. The leaching is probably the result of the bone being in the zone of fluctuating groundwater levels. Remarkably, the ^234^U/^238^U ratios fall within a narrow range of 1.263±0.020 (1-σ standard deviation, excluding the outer point of Tourville E).

Many of teeth provided age estimates (Figure S7.2). In the Figure S7.2, those data points that show clear evidence for leaching (^230^Th/^238^U >> ^234^U/^238^U), a 400 ka data point is shown, where the U-concentrations in the enamel were too low to calculate meaningful age estimates (< 0.2 ppm), a zero age is shown.

In this context one has to keep in mind that U-accumulation takes place after the burial of the bones and teeth, in the first instance, U-series age results provide minimum age estimates. However, as shown above by the human bone samples, a more recent leaching event can either lead to age overestimates or to data sets that prevent any age assessment.

For the teeth in D2 *inf*, the following observations can be made: TOUR1101 shows leaching in the cement, the mean values of the remaining results fall within 80 and 150 ka. The average ^234^U/^238^U ratio is 1.243±0.037. TOUR1102 shows leaching in the outer section of the enamel (which shows a white coloration), the remaining data fall within 150 and 190 ka. The average ^234^U/^238^U ratio is 1.321±0.016. TOUR1104 shows severe leaching in the outer enamel section, the dentine results fall within 150 and 200 ka. The average ^234^U/^238^U ratio is 1.297±0.052. TOUR1105 shows leaching in the outer part of enamel in section B, the remains data of section be are between 150 and 190 ka. The results in section A are significantly older, some well in excess of 250 ka. The average ^234^U/^238^U ratio is 1.343±0.045. TOUR1108 shows leaching in the outer enamel section. The three sections give very different results, section A shows ages raging between 280 and 400 ka, section B between 210 and 310 ka and section C between 215 and 250 ka. The average ^234^U/^238^U ratio is 1.365±0.029.

TOUR1102, TOUR1105 and TOUR1108 have significantly higher ^234^U/^238^U ratios than the other two samples, indicating a different source for the uranium than for TOUR1101 and TOUR1104. Most samples show clear signs of leaching, mostly in the cement as well as the outer layers of the enamel. It is difficult to ascertain whether leaching has taken place in tissues where the ^230^Th/^238^U ratio is smaller than the ^234^U/^238^U ratio. The strong leaching effects in the enamel indicate that the same may just have happened in the adjacent dentine tissues, e.g. in TOUR1108. Thus, it is difficult to use the U-series results for constraining the age of D2 *inf*.

The teeth from D2 *sup* show the following: TOUR1103 seems to show leaching in the cement as well as in the outer section of the enamel. The remaining data fall within 100 and 150 ka. The average ^234^U/^238^U ratio is 1.248±0.037. TOUR1106 shows leaching on the first cement section, all other data fall within 100 to 150 ka The average ^234^U/^238^U ratio is 1.221±0.038. TOUR1107 seems to show leaching in the cement and the remaining data all fall around 100 ka. The average ^234^U/^238^U ratio is 1.247±0.047.

All teeth from D2 *sup* show consistent leaching from the cement. The other data all fall between 100 to 150 ka. The consistent ^234^U/^238^U ratios indicate a similar source for the uranium. The U-series data imply a minimum age for this layer of around 150 ka.

The comparison of the ^234^U/^238^U ratios of the human bone with those of the faunal teeth from D2 *inf* (samples TOUR1101 and TOUR1104) highlights closely similar isotopic signatures. Both teeth also show leaching. Although very different in appearance, TOUR1101 and TOUR1104 show a similar geochemical behavior to the human remains.

4.2. ESR analysis

The D_E_ values are within a relative small range between , i.e. ranging from 121+2 Gy to 175+2 Gy, except for sample TOUR1108 which shows a significantly higher D_E_ value of 254+4 Gy (Table S1). U-concentrations are systematically < 1 ppm for enamel and < 35 ppm for dentine and cement. Radiometric analyses performed on the 4 sediment samples show that radioelement concentrations vary within a narrow range (Table S2): between 0.87 and 1.47 ppm of U, 3.52 and 4.09 ppm for Th, and 0.67 to 0.72 % of K. These values show that the D2 layer is relatively homogenous in terms of radioelement distribution. Three teeth from D2 *inf* (TOUR1104, TOUR1105 and TOUR1108) show the highest apparent U-series ages which precluded any combined US-ESR age calculation for these samples. In contrast, the remaining samples from D2 *sup* show a somewhat distinct pattern. The cement tissues of samples TOUR1106 and TOUR1107 have apparent U-series ages that are significantly older than the other tissues, which again precluded a straight forward US-ESR calculation. Two of the remaining samples (TOUR1101 and TOUR1103) yielded combined US-ESR ages of 184+26/-19 ka and 208 +28/-22 ka, respectively, that are consistent at 1σ (Table 1). The p-values lie overall somewhere between 0.09 and -0.51, i.e. oscillating roughly between EU and LU. The magnitude of the relative errors associated to the age (between 10 and 14 %) is probably mainly explained by the large error in the ^230^Th/^238^U ratios, which are the result of the heterogeneity in the spatial distribution of uranium in the tissues (see Figure S7.2). The difference between the US-ESR age estimates of each sample can be partially explained by lateral variations in the sediment radioactivity.

Sample TOUR1102 did not yield US-ESR results because of the large error in the ^230^Th/^238^U ratios in the enamel which derive from the very low U-concentrations. For this sample we used the dentine U-series data for the enamel as well. In our experience, uranium preferentially migrates into the enamel via the dentine [14-15, 4, 29]. As a result, the enamel ^234^U/^238^U ratios are closely similar to those of dentine and the ^230^Th/^234^U ratios are somewhat smaller. In any case, because of the low U-concentrations in the enamel (< 1ppm), the internal dose rate is a small proportion of the total dose rate so that this assumption presents a negligible source for additional error. The US-ESR age estimation for TOUR1102 agrees within error with the other two samples (Table 1). The weighted mean of the US-ESR calculations derived from the three samples is 194+14/-11 ka.

As explained above, the CSUS-ESR ages are somewhat older but nevertheless consistent with the standard US-ESR ages, and may vary between 188+21 and 236+29 ka (Table 1). The weighted mean of the CSUS-ESR calculations is 211±15 ka. Thus the best age range estimate for the teeth derives from the error envelope given by both models, 183 to 226 ka, which represents the end of MIS 7 to the beginning of MIS6.

**Text S5. Preservation of the Tourville fossils**

The 232 mm long portion of the left humerus is represented by its diaphysis and the supero-lateral portion of the distal epiphysis. The diaphysis is complete from the surgical neck region to its distal third. Distally only several fragments of the anterior surface and a piece of the postero-medial surface are preserved. The medial supra-condylar ridge and the summit of the olecranon fossa are both present on the distal extremity.

Although the proximal two-thirds of the left ulna is preserved and measures 150 mm in length, reliably approximating the length of the complete bone is not possible. The ulna is broken below the proximal articular extremity and a few centimetres above the distal extremity. On the anterior surface, close to the interosseous border, a long and deep depression is visible that could correspond to the main nutrient foramen.

The preserved portion of the left radius is 146 mm long and represents the proximal three-quarters of the diaphysis. The proximal break occurs at the top of the radial neck, while the distal part is located at the level of the lower third of the diaphysis.

**Text S6. Comparison groups used in the morphometric analysis**

The first comparison group comprises recent anatomically modern humans compiled by Hambucken [30], while the second and third are composed of members of the Neandertal line, which we have divided into two groups: Middle and early Late Pleistocene (MIS 5e), commonly referred to as 'Early Neandertals' [31] but considered here as 'Pre-Neandertals' (PNEAND), and a second group comprising Neandertal fossils from colder and more recent phases of the Late Pleistocene (NEAND). The Pre-Neandertal sample contains fossils (e.g. the Mauer or the Sima de los Huesos specimens; [32-34] that certain researchers attribute to *Homo heidelbergensis* (but see also [35-36]. We follow Hublin [37] or Stringer [36] in considering the European fossils post-dating MIS 11 and bearing derived features (metrical or non-metrical) of Neandertals to be members of a line closely related to the Neandertals.

Humerus cortical bone thickness was studied using chromatic scaling [38-39] and compared with a Neandertal humerus (Krapina 165) from the NESPOS database [40].

**Text S7. CT-scan methodology and results**

The Tourville-la-Rivière human remains were CT-scanned at the University Hospital Pellegrin in 2012 using an Optima CT660 (GE Medical Systems). Their final volumes were reconstructed in a 16-bit format with a 0.17×0.17×0.7 mm/voxel resolution. A semi-automatic threshold-based segmentation with manual corrections was carried out using Avizo v.7 (Visualization Sciences Group) following the half maximum height method [41] adapted to 3D data [42]. Surface rendering was performed using triangulation and constrained smoothing from the volumetric data (marching cube algorithm; [43]). Three-dimensional topographic mapping of the cortical thickness variation was rendered by a chromatic scale, where bone thickness is defined for each point on the periosteal surface as the distance to the closest point on the endosteal surface (thickness increasing from dark blue up to red; e.g. [39]).

The results of the virtual analysis of the left humerus from Tourville-la-Rivière (A) were compared to CT-based evidence from the adult Neandertal specimen Krapina 165 (B), Croatia (NESPOS database, [40]). Important differences are visible in terms of cortical thickness between the two specimens on the posterior, medial, and lateral surfaces. This may result either from post-mortem erosion of the diaphysis, particularly in regards to the posterior and lateral views, and or the inter-individual variability of the Pre-Neandertals, with the Tourville specimen having a less pronounced cortical thickness. However, in all three views, the crest, which corresponds to an enthesopathy of the posterior deltoid muscle insertion, is clearly visible in the form of localised thickening of the cortical bone.

**References**

1. Boëda E (1990) De la surface au volume : analyse des conceptions des débitages Levallois et laminaire. In: C. Farizy, editor. Paléolithique Moyen Récent et Paléolithique Supérieur Ancien en Europe. Mém Mus Préh Ile de Fr 3: 63-68.

2. Otte M (1994) Rocourt (Liège, Belgique): industrie laminaire ancienne. In: S. Revillion and A. Tuffreau editors. Les industries laminaires au Paléolithique Moyen. Doss Doc Archéo 18:180-186.

3. Aubert M, Pike AWG, Stringer C, Bartsiokas A, Kinsley L, et al. (2012) Confirmation of a late Middle Pleistocene age for the Omo Kibish 1 cranium by direct uranium-series dating. J Hum Evol 63: 704-710.

4. Grün R, Aubert M, Joannes-Boyau R, Moncel MH (2008) High resolution analysis of uranium and thorium concentrations as well as U-series isotope distributions in a Neanderthal tooth from Payre using laser ablation ICP-MS. Geo Cosmo Acta 72: 5278-5290.

5. Mijares AS, Détroit F, Piper P, Bellwood P, Grün R, et al. (2010) New evidence for a 67,000 year old human presence at Callao Cave, Luzon, Philippines. J Hum Evol 59**:** 123-132.

6. Storm P, Grün R, Stringer C, Bartsiokas A, de Vos J, et al. (2013) New dates for "Wajak Man" (Java, Indonesia) suggest a Late Pleistocene age. J Hum Evol 64: 356-365.

7. Millard AR (1993) Diagenesis of archaeological bone: the case of uranium uptake. D.Phil. Thesis, University of Oxford.

8. Millard AR, Hedges REM (1996) A diffusion-adsorption model of uranium uptake by archaeological bone. Geo Cosmo Acta 60: 2139-2152.

9. Pike AWG (2000) Uranium series dating of archaeological bone by thermal ionization mass spectrometry. D.Phil. Thesis, University of Oxford, Oxford.

10. Pike AWG, Hedges REM, Van Calsteren P (2002) U-series dating of bone using the diffusion-adsorption model. Geo Cosmo Acta 66: 4273-4286.

11. Sambridge M, Grün R, Eggins S (2012) U-series dating of bone in an open system: The diffusion-adsorption-decay model. Quat Geochrono 9: 42-53.

12. Grün R, Schwarcz HP, Chadam J (1988) ESR dating of tooth enamel: coupled correction for U-uptake and U-series disequilibrium. Nuclear Tracks and Radiation Measurements 14: 237-241.

13. Grün R (2000a) An alternative for model for open system U-series/ESR age calculations: (closed system U-series)-ESR, CSUS-ESR. Ancient TL 18: 1-4.

14. Eggins SM, Grün R, Pike A, Shelley A, Taylor L (2003) ^238^U, ^232^Th profiling and U-series isotope analysis of fossil teeth by laser ablation ICPMS. Quat Sci Rev 22: 1373-1382.

15. Eggins SM, Grün R, McCulloch MT, Pike AWG, Chappell J, et al. (2005) In situ U-series dating by laser-ablation multi-collector ICPMS: new prospects for Quaternary geochronology. Quat Sci Rev 24: 2523-2538.

16. Longerich HP, Jackson SE, Gunther D (1996) Laser ablation inductively coupled plasma mass spectrometric transient signal data acquisition and analyte concentration calculation. J. Anal. At. Spectrom. 11: 899–904.

17. Grün R, Huang PH, Huang W, McDermott F, Stringer CB, et al. (1998) ESR and U-series analyses of teeth from the palaeoanthropological site of Hexian, Anhui Province, China. J Hum Evol 34: 555-564.

18. Duval M, Falguères C, Bahain JJ, Grün R, Shao Q, et al. (2011a) The challenge of dating Early Pleistocene fossil teeth by the combined uranium series–electron spin resonance method: the Venta Micena palaeontological site (Orce, Spain). J Quat Sci 26: 603-615.

19. Grün R, Rhodes EJ (1992) Simulations of saturating exponential ESR/TL dose response curves - weighting of intensity values by inverse variance. Ancient TL 10: 50-56.

20. Grün R (2000b) Methods of dose determination using ESR spectra of tooth enamel. Radiation Measurements 32: 767-772.

21. Grün R, Brumby S (1994) The assessment of errors in past radiation doses extrapolated from ESR/TL dose-response data. Radiation Measurements 23: 307-315.

22. Duval M, Grün R, Falguères C, Bahain JJ, Dolo JM (2009) ESR dating of Lower Pleistocene fossil teeth: Limits of the single saturating exponential (SSE) function for the equivalent dose determination. Radiation Measurements 44: 477-482.

23. Grün R (2009) The DATA program for the calculation of ESR age estimates on tooth enamel. Quat Geochrono 4: 231-232.

24. Grün R, Katzenberger-Apel O (1994) An alpha irradiator for ESR dating. Ancient TL 12: 35-38.

25. Marsh RE (1999) Beta-gradient isochrons using electron paramagnetic resonance: towards a new dating method in archaeology. MSc thesis, McMaster University, Hamilton.

26. Guérin G, Mercier N, Adamiec G (2011) Dose-rate conversion factors: update. Ancient TL 29: 5-8.

27. Prescott JR, Hutton JT (1988) Cosmic ray and gamma ray dosimetry for TL and ESR. Nuclear Tracks and Radiation Measurements 14: 223-227.

28. Prescott JR, Hutton JT (1994) Cosmic ray contributions to dose rates for luminescence and ESR dating: Large depths and long-term time variations. Radiation Measurements 23: 497-500.

29. Duval M, Aubert M, Hellstrom J, Grün R (2011b) High resolution LA-ICP-MS mapping of U and Th isotopes in an early Pleistocene equid tooth from Fuente Nueva-3 (Orce, Andalusia, Spain). Quat Geochrono 6: 458-467.

30. Hambucken A (1993) Variabilité morphologique et métrique de l’humérus, du radius et de l’ulna des Néandertaliens. Comparaison avec l’Homme moderne. Thèse, Université Bordeaux 1, 302 p.

31. Dean D, Hublin JJ, Holloway R, Ziegler R (1998) On the phylogenetic position of the pre-Neandertal specimen from Reilingen, Germany. J Hum Evol 34: 485-508.

32. Rosas A, Bermúdez De Castro JM (1998) The Mauer mandible and the evolutionary significance of *Homo heidelbergensis*. Geobios, 31(5): 687-697.

33. Bermúdez de Castro JM, Martinón-Torres M, Sarmiento S, Lozano M (2003) Gran Dolina-TD6 versus Sima de los Huesos dental samples from Atapuerca: evidence of discontinuity in the European Pleistocene population? J Archaeol Sci 30: 1421-1428.

34. Mounier A, Marchel F, Condemi S (2009) Is *Homo heidelbergensis* a distinct species? New insight on the Mauer mandibule. J Hum Evol 56: 219-246.

35. Arsuaga JL, Bermúdez De Castro JM, Carbonell E (1997) The Sima de los Huesos site. J Hum Evol 33: 105-421.

36. Stringer CB (2012) The Status of *Homo heidelbergensis* (Schoetensack 1908). Evol Anthropol 21: 101-107.

37. Hublin JJ (2009) The origin of Neandertals. Proc Natl Acad Sci USA 106:16022-16027.

38. Bondioli L, Bayle P, Dean C, Mazurier A, Puymerail L, et al. (2010) Technical note: morphometric maps of long bone shafts and dental roots for imaging topographic thickness variation. Am J Phys Anthropol 142: 328-334.

39. Puymerail L, Volpato V, Debénath A, Mazurier A, Tournepiche JF, et al. (2012) A Neanderthal partial femoral diaphysis from the “grotte de la Tour”, La Chaise-de-Vouthon (Charente, France): Outer morphology and endostructural organization. C R Palevol 11(8): 581-593.

40. NESPOS Database (2013) Neanderthal studies professional online service. [http://www.nespos.org](http://www.nespos.org/).

41. Spoor F, Zonneveld F, Macho GA (1993) Linear measurements of cortical bone and dental enamel by computed tomography: applications and problems. Am J Phys Anthropol 91: 469-484.

42. Coleman MN, Colbert MW (2007) CT thresholding protocols for taking measurements on three-dimensional models. Am J Phys Anthropol 133: 723-725.

43. Lorensen WE, Cline HE (1987) Marching cubes: A high resolution 3D surface construction algorithm. Comput Graph (ACM) 21:163-169.

44. Trinkaus E, Churchill S (1999) Diaphyseal cross-sectional geometry of Near Eastern Middle Palaeolithic humans: the humerus. J Archaeol Sci 26: 173-184.

45. Vandermeersch B (1981) Les Hommes fossiles de Qafzeh (Israël). Cahiers de Paléontologie. Paris : éds. du C.N.R.S. 319 p.

46. Heim JL (1982) Les hommes fossiles de La Ferrassie, vol. 2 : Les squelettes adultes (squelette des membres). Paris, Masson, 280 p.

47. Trinkaus E, Churchill SE, Ruff CB (1994) Postcranial robusticity in *Homo*, II: Humeral bilateral asymmetry and bone plasticity. Am J Phys Anthropol 93: 1-34.

48. Walker J, Ortega J, López MV, Parmová K, Trinkaus E (2011) Neandertal Postcranial Remains From the Sima de las Palomas del Cabezo Gordo, Murcia, Southeastern Spain. Am J Phys Anthropol 144: 505-515.

49. Vandermeersch B, Trinkaus E (1995) The postcranial remains of the Regourdou 1 Neandertal: The shoulder and arm remains. J Hum Evol 28: 439-476.

50. Trinkaus E (1983). The Shanidar Neandertals. New York: Academic Press, 502 p

51. McCown T, Keith A (1939) The Stone Age of Mount Carmel. The fossil human remains from the Levalloiso-Mousterian, vol. II. Oxford: Clarenton Press, 390 p.

52. Carretero JM, Arsuaga JL, Lorenzo C (1997) Clavicles, scapulae and humeri from the Sima de los Huesos site (Sierra de Atapuerca, Spain). J Hum Evol 33: 357-408.

53. Carretero JM, Lorenzo C, Arsuaga JL (1999) Axial and appendicular skeleton of *Homo antecessor*. J Hum Evol 37: 459-499.

54. Martin R (1914) Lehrbuch der Anthropologie Jena: Gustav Fischer, pp. 475-890.

55. Maureille B, Rougier H, Houet F, Vandermeersch B (2001) Les dents inférieures du néandertalien Regourdou 1 (site de Regourdou, commune de Montignac, Dordogne) : analyses métriques et comparatives. Paléo 13: 183-200.

56. Scolan H, Santos F, Tillier AM, Maureille B, Quintard A (2012) Des nouveaux vestiges néanderthaliens à Las Pélénos (Monsempron-Libos, Lot-et-Garonne, France). Bull. Mém. Soc. Anthropol. Paris 24: 69-95.

**Supporting Figures (Figure S1 – Figure S11)**

**Figure S1.**

Spatial distribution of the faunal remains.

**Figure S2.**

The D2 *inf* faunal assemblage

**Figure S3.**

Spatial distribution of lithic artefacts and focus on the knapping area.

**Figure S4.**

Refitting sequence comprising 46 pieces from the knapping concentration (a). While most elements of the reduction sequence are represented (waste, core management and shaping flakes, fragments of flakes and blades), several refitting sequences (b and c) show that the cores and largest products were exported.

**Figure S5.**

Rocourt-type debitage. 1- Elongated *éclats débordants* refit with laminar flake fragments. The negatives evince a bipolar debitage method producing either laminar flakes or blades. 2, 3 – Rocourt-type blades.

**Figure S6.**

Examples of macro-wear (scarring) on Levallois products probably used to work soft animal materials.

**Figure S7.1.**

U-series results of five bone fragments of the human remains. Top left: sample holder before analysis, left column: laser ablation analysis spots (the spot diameters are around 250 μm across); right column: U-series isotope results. When the ^230^Th/^238^U ratio is > ^234^U/^238^U then leaching has occurred and no age can be calculated.

**Figure S7.2.**

U-series results on eight faunal teeth. Left: photos on the cross sections with laser ablation pits. The arrows indicate the analysis direction. Middle column: U-series isotope results. Right column: apparent U-series age estimates. Leaching is indicated by 400 ka age estimates, U-concentrations too low for age calculation are shown as zero ages.

**Figure S8.**

Schematic representation of the adjusted Z-scores for Tourville humerus relative to Pre-neandertal (blue curve), Neandertal (red curve), and extant modern human variability (green curve). Dmax = maximal diameter at mid-diaphysis; Dmin = minimal diameter at mid-diaphysis (M6); P6/12 = Perimeter at mid-diaphysis (M7a); P5/12 = Perimeter at the level of the deltoid tuberosity; INDTub = [(P5/12)/(P6/12)*100]; INDia = [(Dmax/Dmin)*100]

**Figure S9.**

Comparison of the deltoid lateral crest insertion to (A) the left humerus of La Sima de los Huesos humerus III (anterior view) and (B) the crest (lateral view) from Carretero et al. [52]. Close-up (C) of the Tourville specimen (lateral view). Dotted line: orientation of the crest.

**Figure S10.**

Schematic representation of the adjusted Z-scores of the Tourville ulna relatively to the Neandertal variability (blue curve) and extant modern humans (green curve). Same legend as table S4.

**Figure S11.**

Schematic representation of the adjusted Z-scores of the Tourville radius relatively to the Preneandertal variability (blue curve), Neandertal variability (red curve) and extant modern humans (green curve). Same legend as table S5.

**Supporting Tables (Table S1 – Table S7)**

**Table S1.**

U-series and ESR data obtained for all the Tourville samples. E = enamel, D = dentine, C = cement. Standard errors are expressed at 1 σ. (1): outer part of the enamel (i.e. cement side); (2): inner side of the enamel (i.e. dentine side).

**Table S2.**

Radioelement concentration obtained for the sediment. In italics, samples for which the sediment was collected from the same layer, but not in direct contact with the tooth.

**Table S3.**

Specimens used for comparing the Tourville upper limb dimensions and the cross-section properties of the humerus.

x = metric; xx = metric and cross-sectional geometric properties

**Table S4.**

Dimensions of the Tourville humerus.

Dmax = maximal diameter at mid-diaphysis ; Dmin = minimal diameter at mid-diaphysis (M6); P6/12 = Perimeter at mid-diaphysis (M7a); P5/12 = Perimeter at the level of the deltoid tuberosity; INDTub = [(P5/12)/(P6/12)*100]; INDia = [(Dmax/Dmin)*100];

L = left, R = right, m = mean, s = standard deviation, n = size of the sample; PNEAND = Preneandertal sample, NEAND = Neandertal sample, RMH : extant human sample; TOUR-PNEAND = adjusted Z-score of Tourville relative to the Preneandertal sample; TOUR-NEAND = adjusted Z-score of Tourville relative to the Neandertal sample; TOUR-RMH = adjusted Z-score of Tourville relative to the extant modern human sample.

The measurements of Tabun 1 are provided as an indication of a gracile individual.

Adjusted Z-scores have been computed to compare Tourville to the range of variation of each comparative group.

Definition of the diameters/perimeters following Martin [54] and Hambucken [30].

The adjusted z-scores have been defined following Maureille et al [55] and Scolan et al [56]. All the necessary explanations for the correspondence between adjusted z-scores and standard deviations of the comparative groups are available in these articles.

**Table S5.**

Cross-sectional geometric properties of the Tourville humerus and comparison with the Tabun C1 and Neandertal sample (NEAND ; cf. Table S3).

TA = Subperiostal area, %CA = Relative cortical thickness, %MA = Relative medullar area, Ix/Iy = Diaphyseal circular index, J = Polar second moment of area, 50 = section at mid-diaphysis, 65 = section at 65% of the diaphysis.

L = left, R = right, m = mean, s = standard deviation, n = sample size.

The cross-sectional geometric properties have been taken at 50% and 65% of the diaphysis.

**Table S6.**

Dimensions of the Tourville ulna. Same legend as table S4.

Dap1/2 = antero-posterior diameter at mid-diaphysis (M11); Dtr1/2 = Transversal diameter at mid-diaphysis (M12); INDia = [((Dap1/2)/(Dtr1/2))*100].

r = range of variation.

For the definition of the diameters/perimeters and adjusted z-scores, see table S4.

**Table S7**.

Dimensions of the Tourville radius. Same legend as table S4.

Ptb = Perimeter of the diaphysis below the distal tuberosity; P1/2 = Perimeter at mid-diaphysis (M5(5)); Dtr1/2 = Transversal Diameter at mid-diaphysis (M4a); Dap1/2 = antero-posterior diameter at mid-diaphysis (M5a); DtrC = Transversal interosseous diameter (M4); DapC = Antero-posterior interosseous diameter (M5); INDia = [((Dap1/2)/(Dtr1/2))*100]; INDC = [(DapC/DtrC)*100].

For the definition of the diameters/perimeters and adjusted z-scores, see table S4.


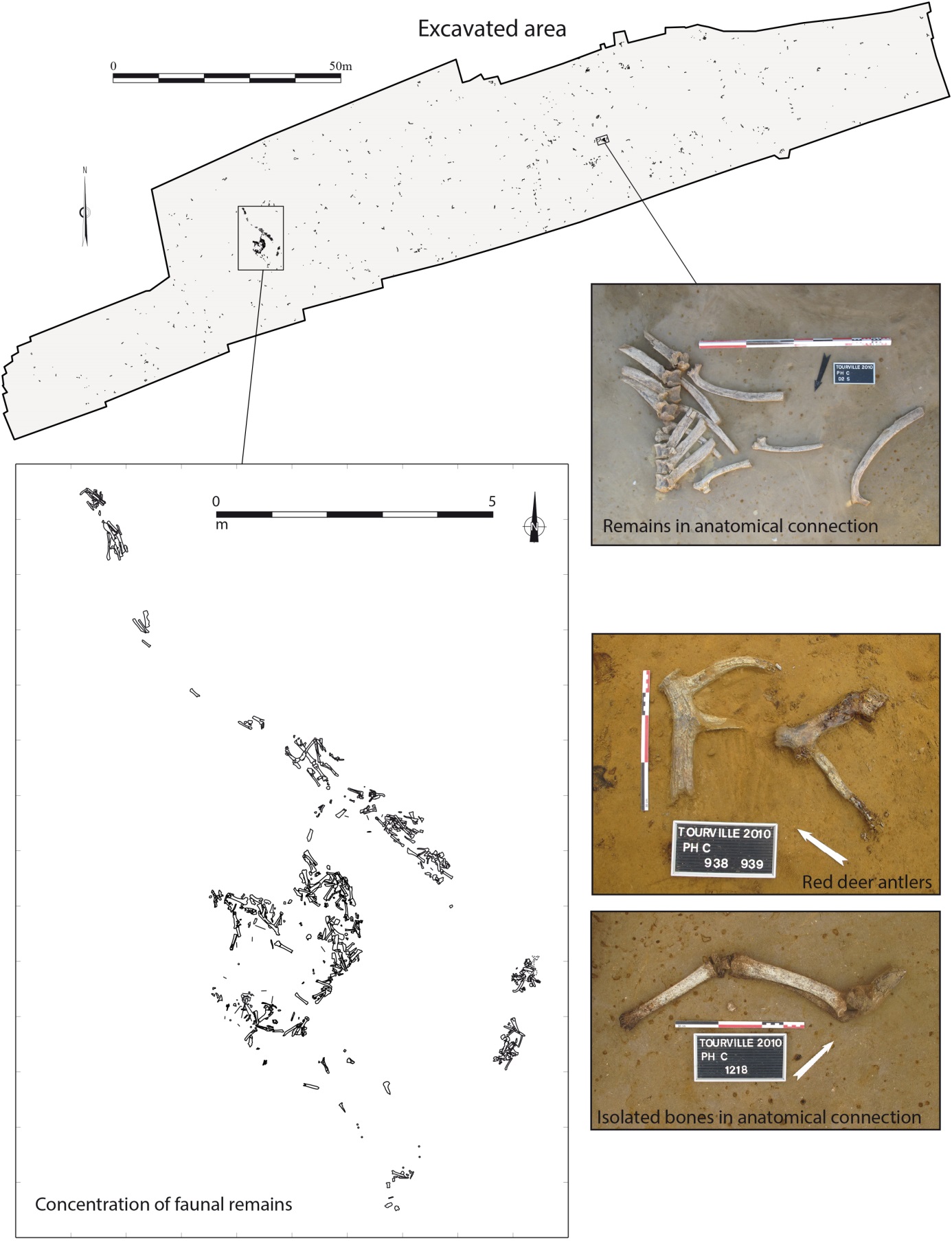


Figure S1.


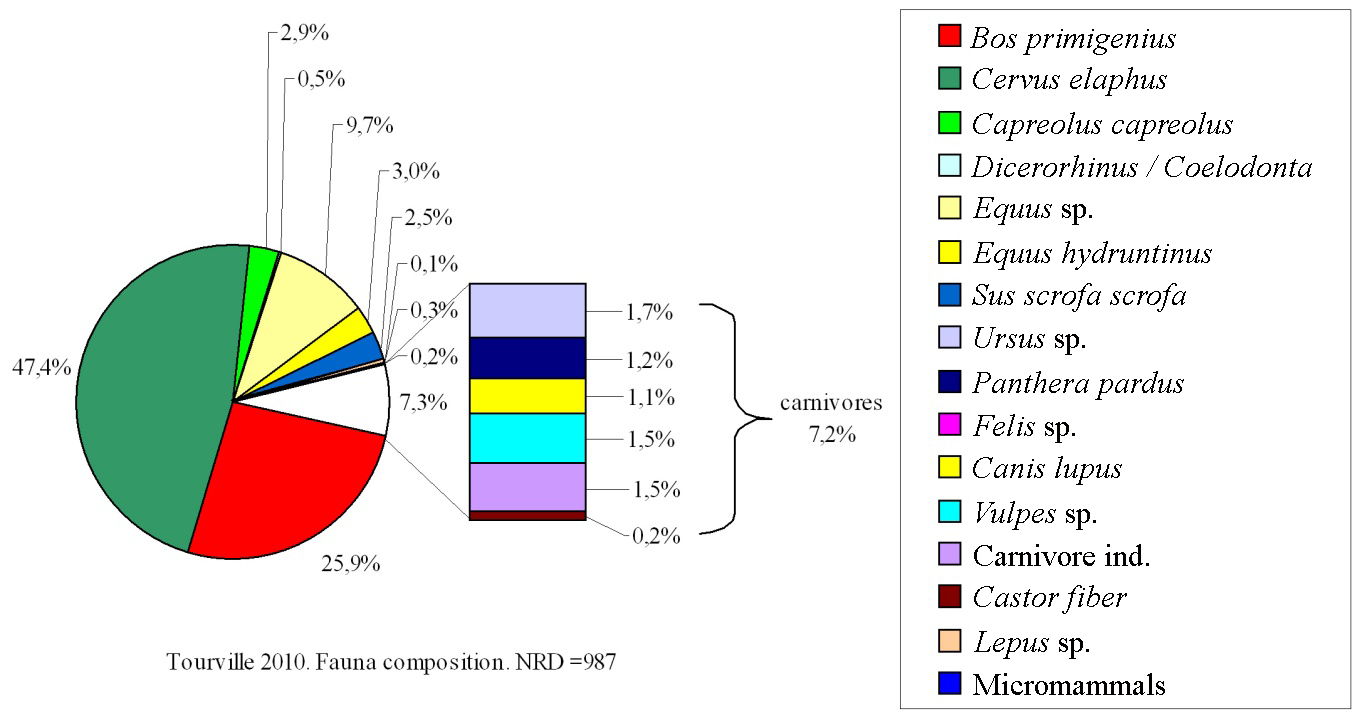


Figure S2.

**
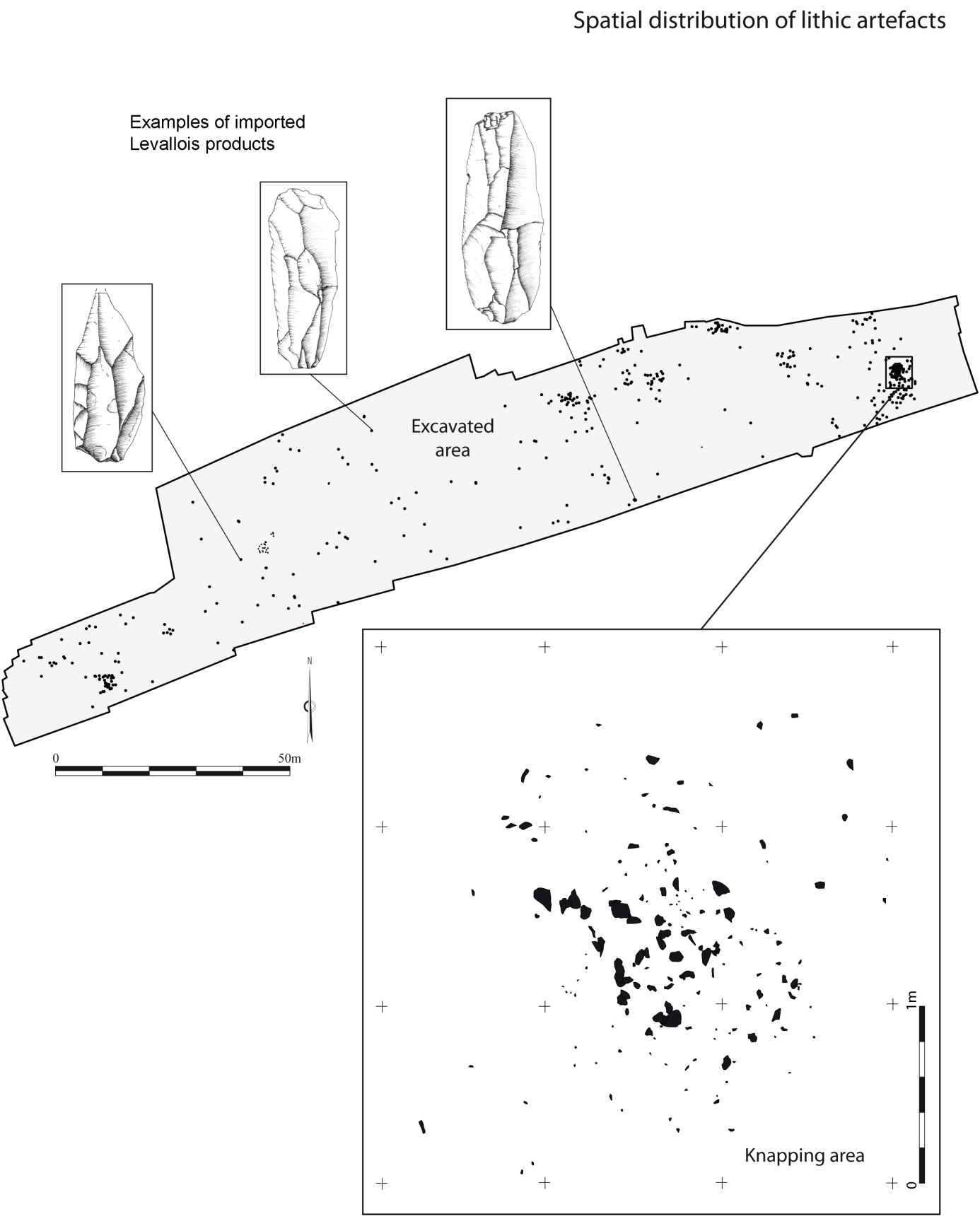
**

Figure S3.


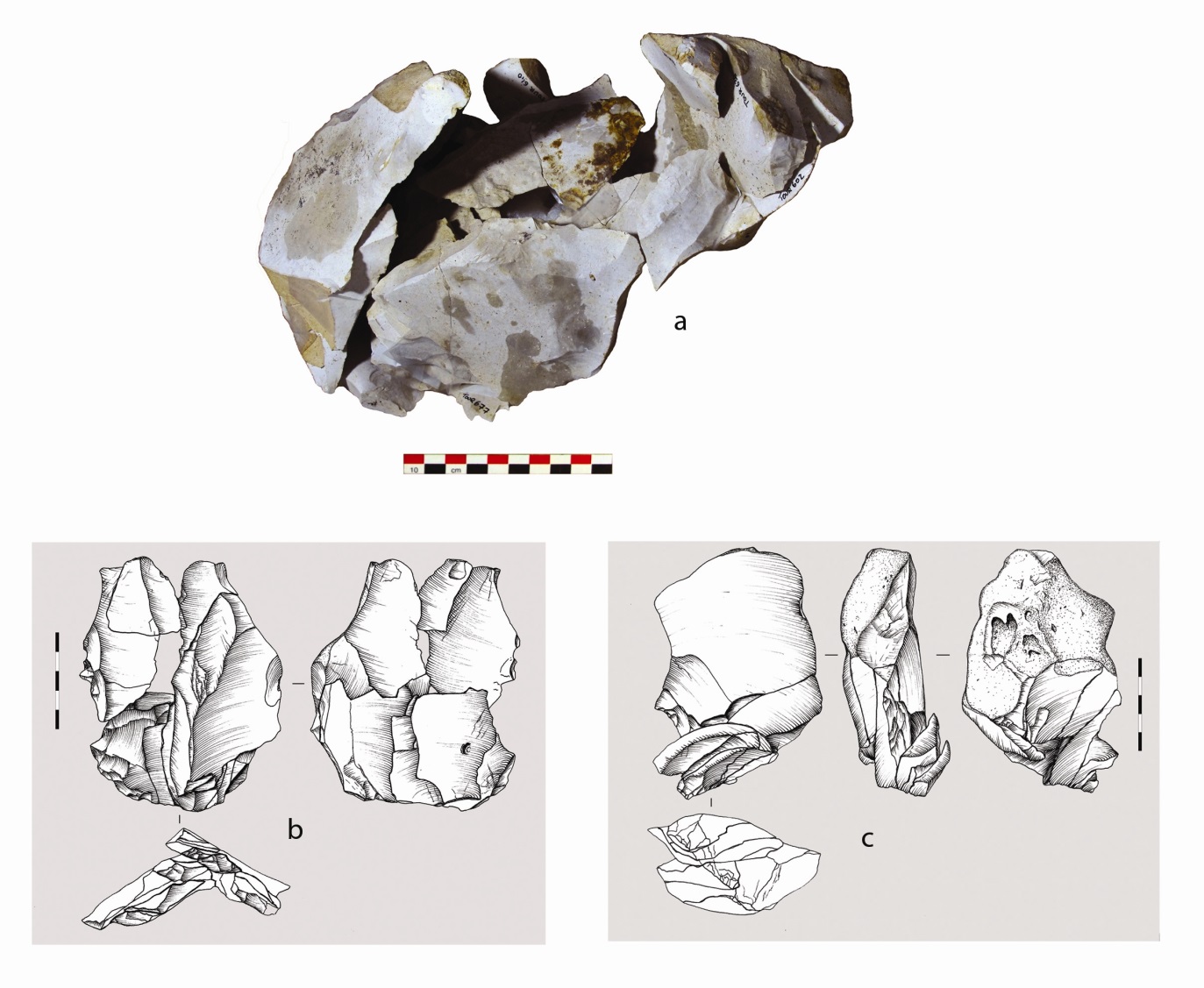


Figure S4.


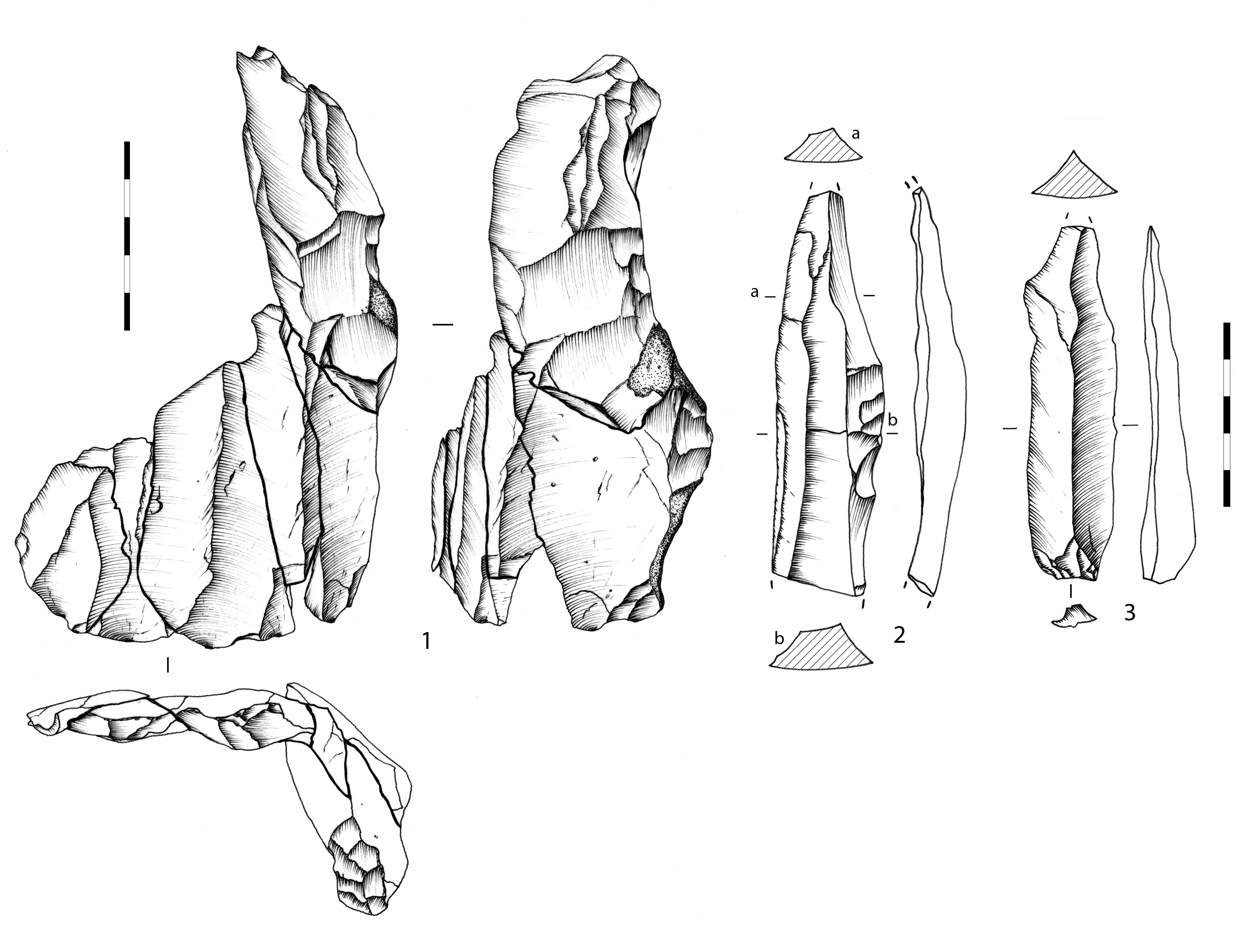


Figure S5.


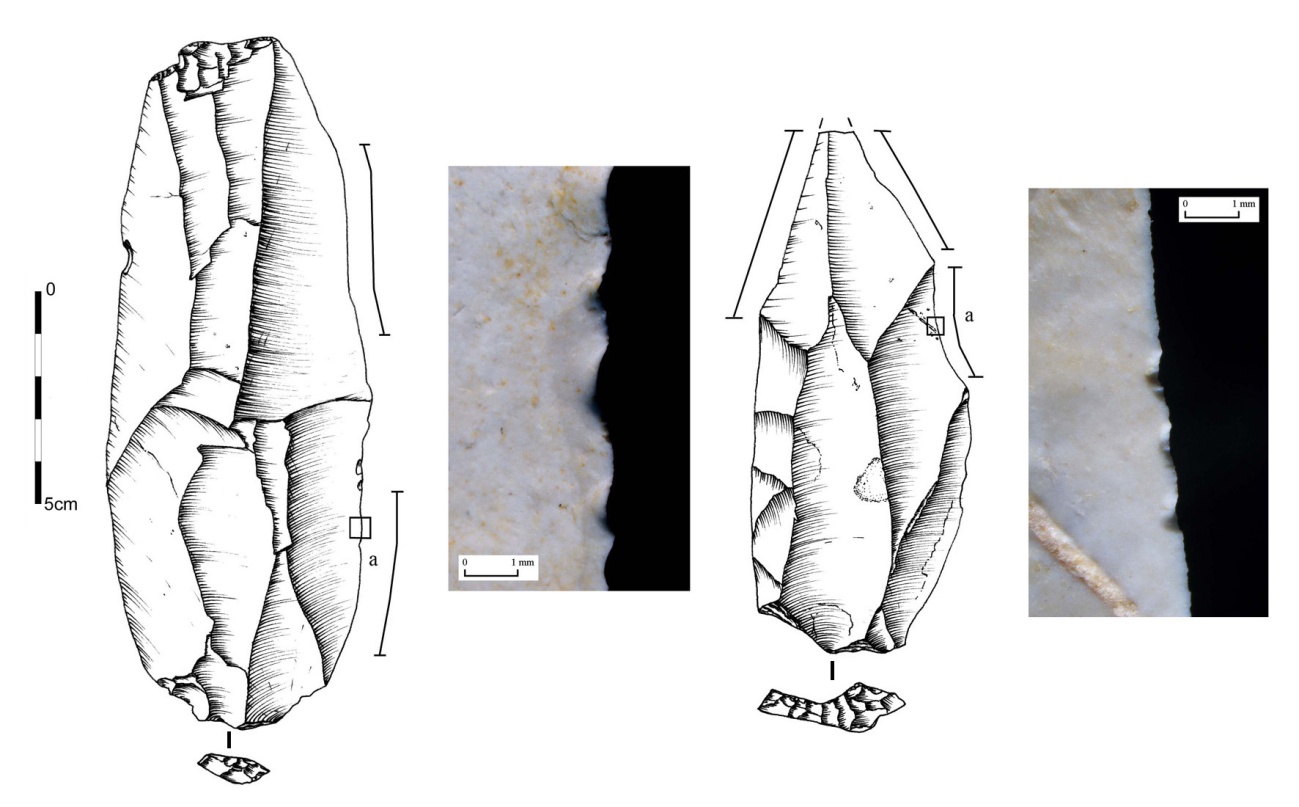


Figure S6.


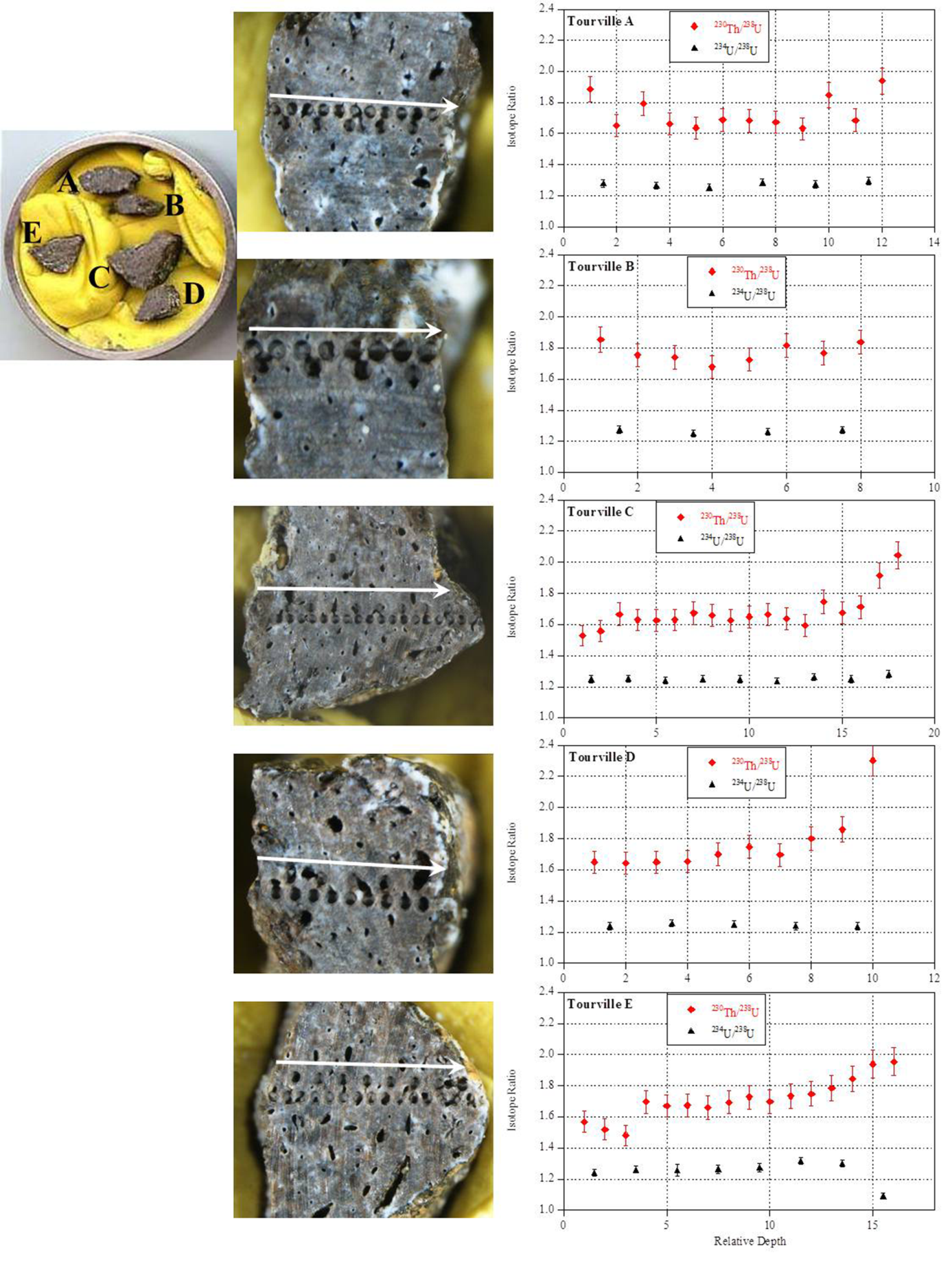


Figure S7.1.


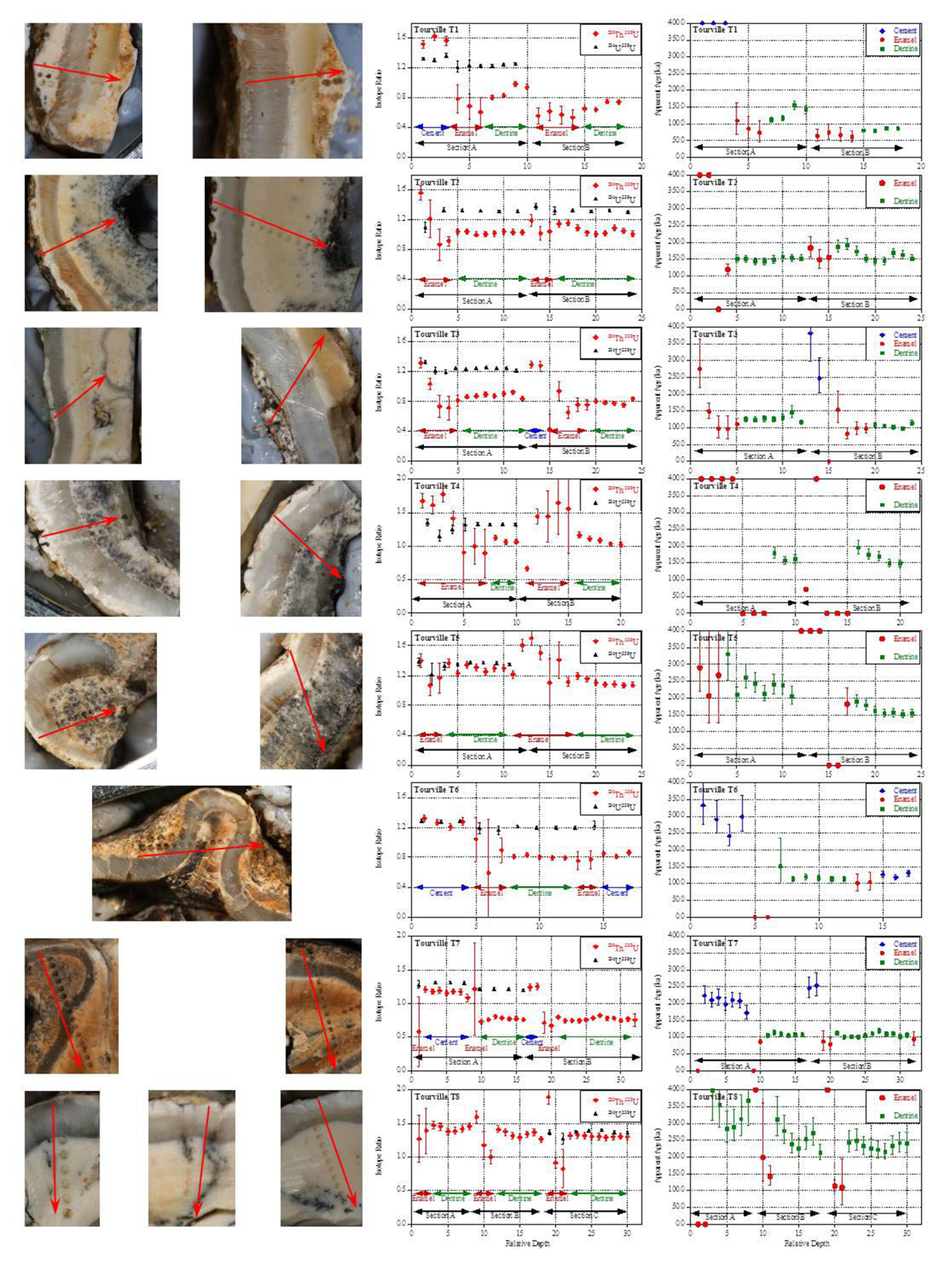


Figure S7.2.


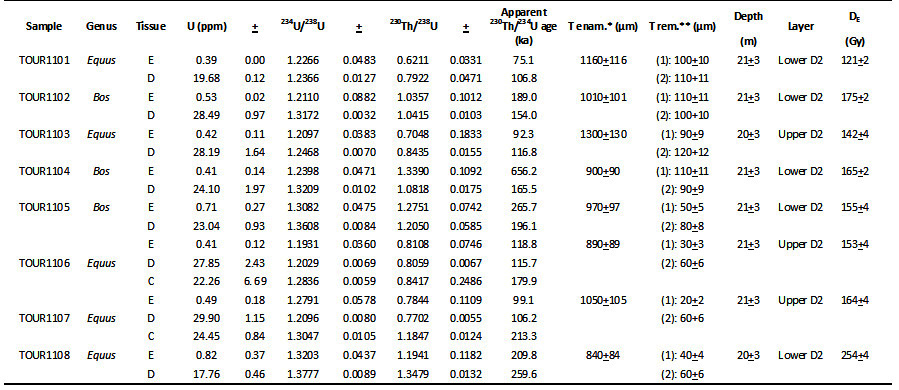


Table S1.

Table S2.

|  | **HUMERUS** | **RADIUS** | **ULNA** | **References** |
| --- | --- | --- | --- | --- |
| *NEAND: Late Pleistocene Neandertals* |  |  |  |  |
| Amud 1 | x | x | x | [44-45] |
| Feldhofer | xx | x | x | [46-47] |
| Kebara 2 | xx | x |  | [47,44]; Cast |
| La Chapelle-aux-Saints 1 | xx | x | x | Original; [47] |
| La Ferrassie 1 & 2 | xx | x | x | [46-47] |
| La Quina 5 | xx |  | x | [46-47] |
| Las Palomas 16, 64, 92 | xx | x | x | [48] |
| Lezetxiky | xx |  |  | [47] |
| Regourdou | xx | x | x | [49] |
| Shanidar 1, 3, 4, 6 & 8 | xx | x | x | [50, 47, 44] |
| Spy I & II | xx | x | x | Original; [47] |
| Tabun 1 | xx | x | x | [51, 47, 44]; Cast |
| *PNEAND: Middle Pleistocene Neandertals* |  |  |  |  |
| Sima de los Huesos | x | x |  | [52-53] |
| Krapina | x | x | x | Original |
| *RMH: Recent Modern Humans* |  |  |  |  |
| Worldwide populations | x | x | x | Original; [30] |

Table S3.


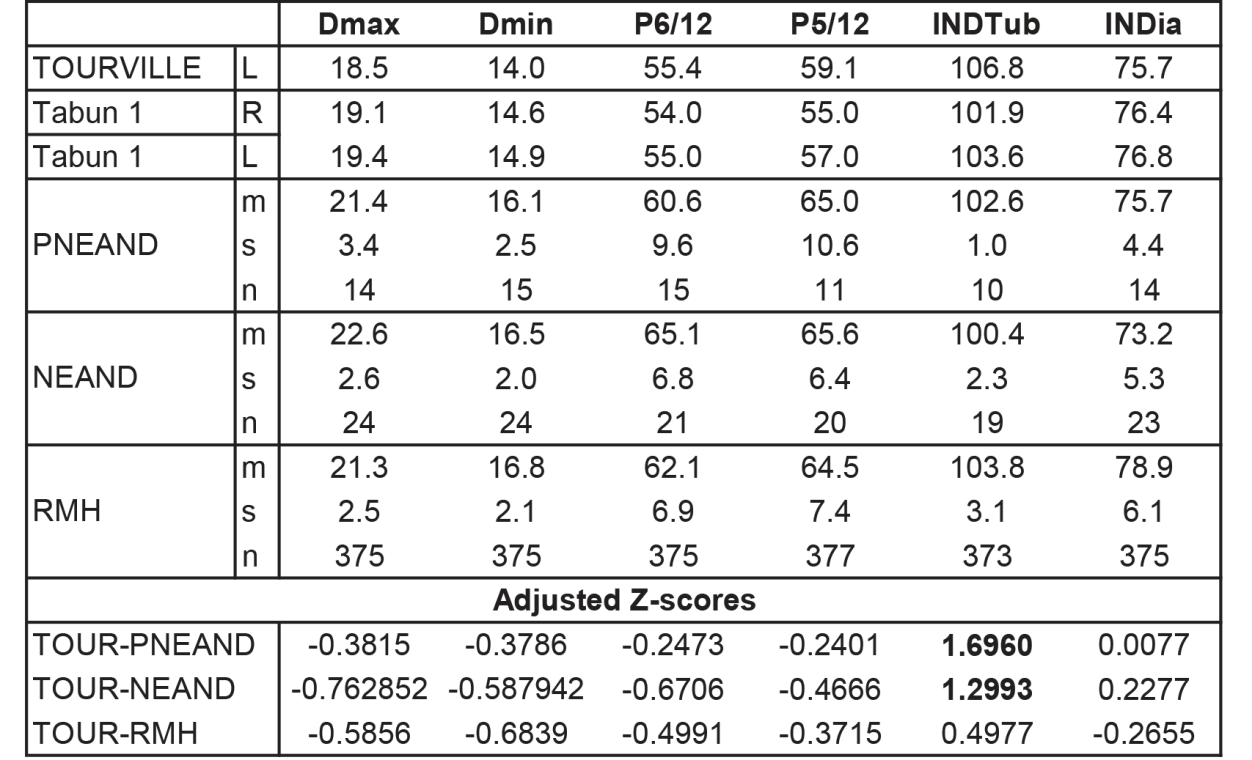


Table S4.


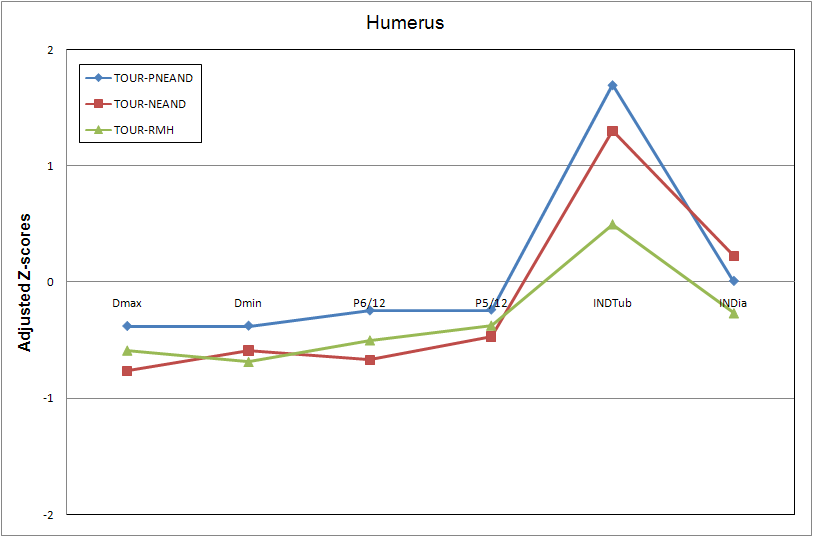


Figure S8.


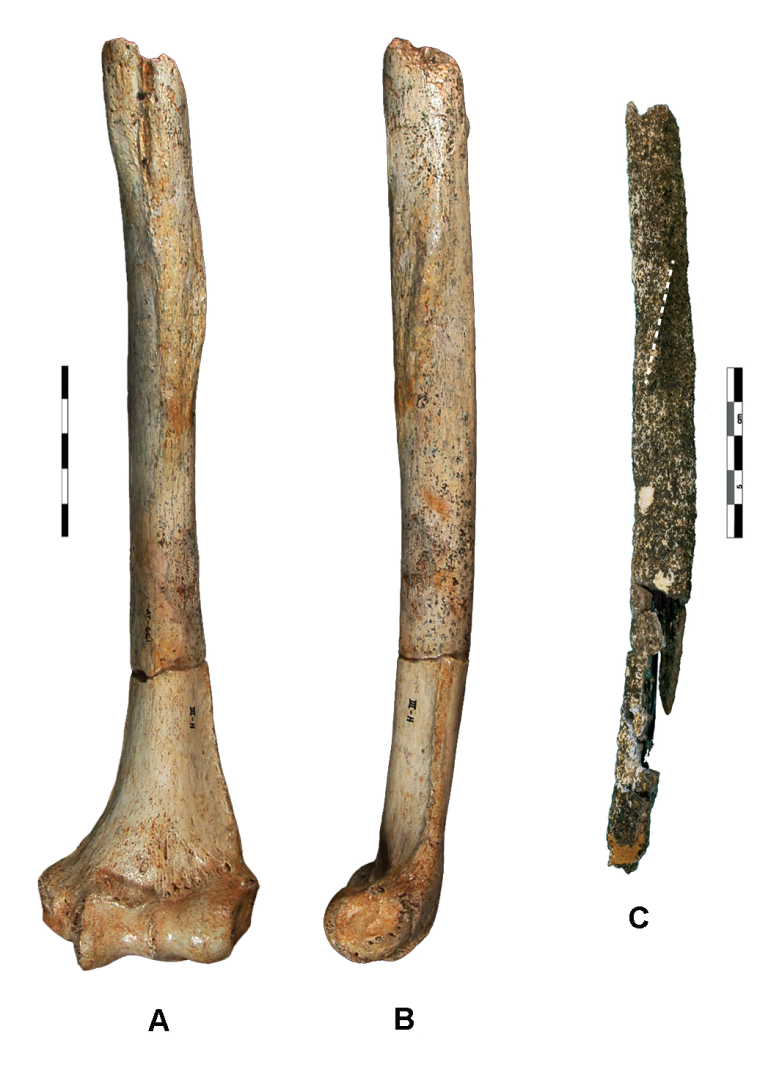


Figure S9.


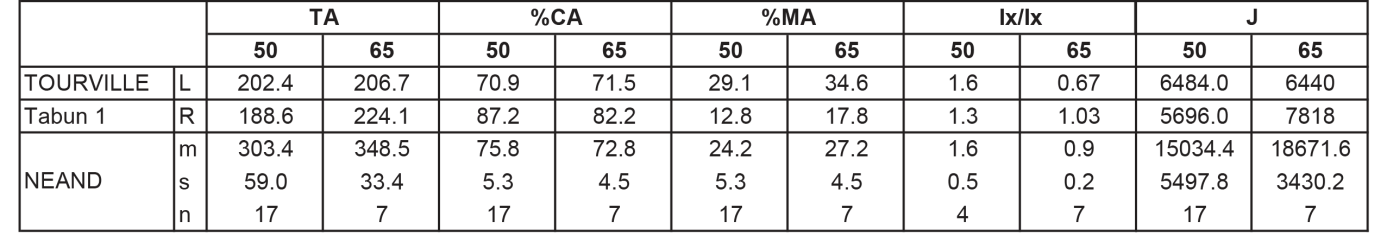


Table S5.


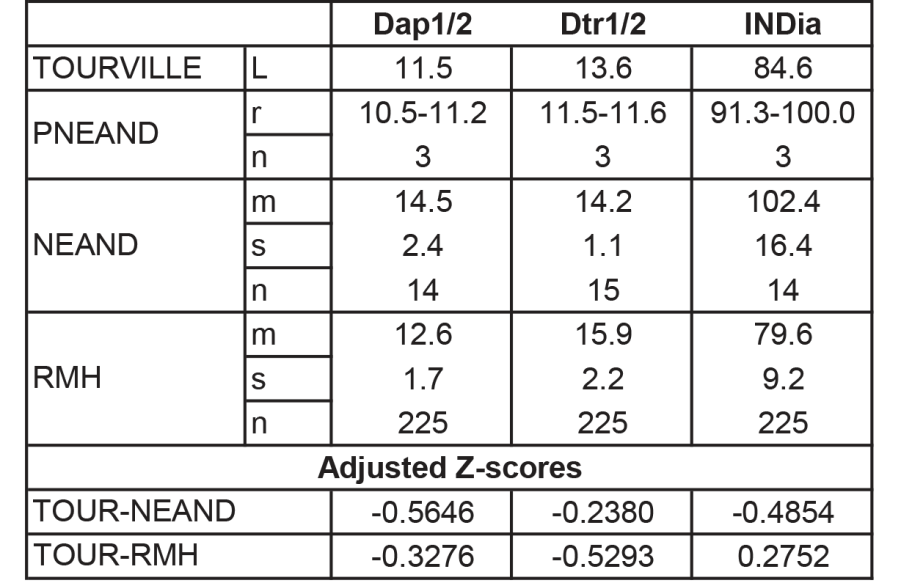


Table S6.


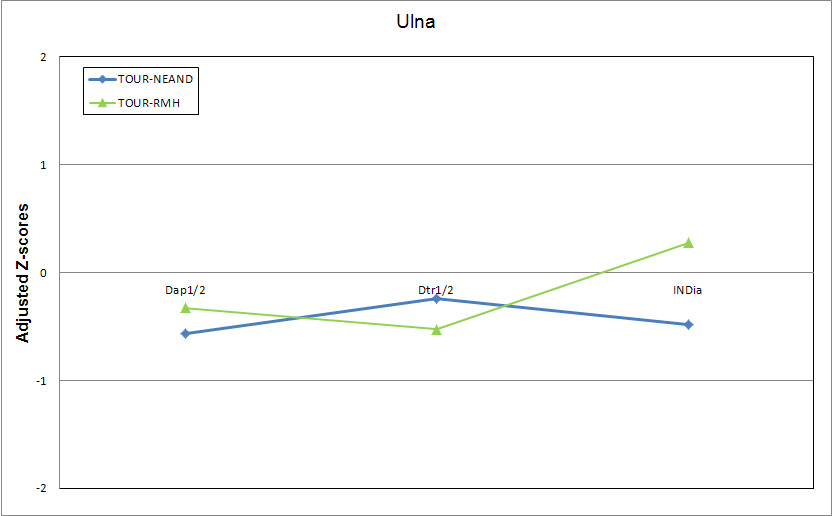


Figure S10.


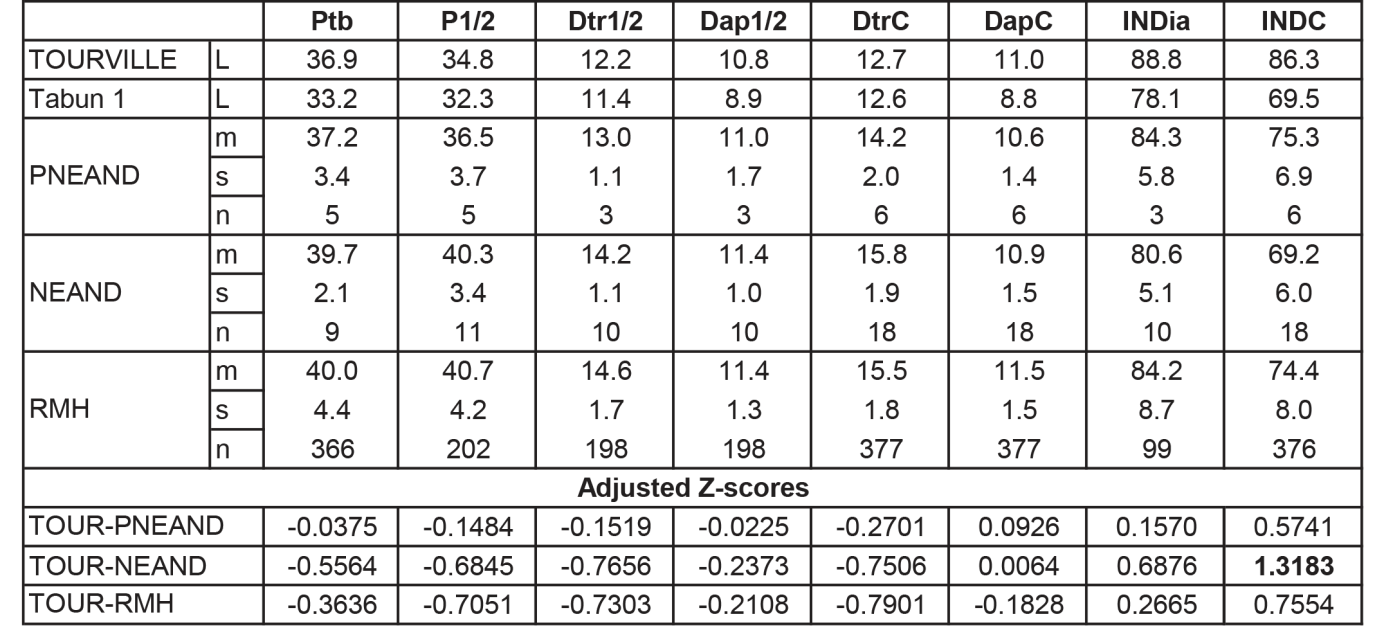


Table S7.


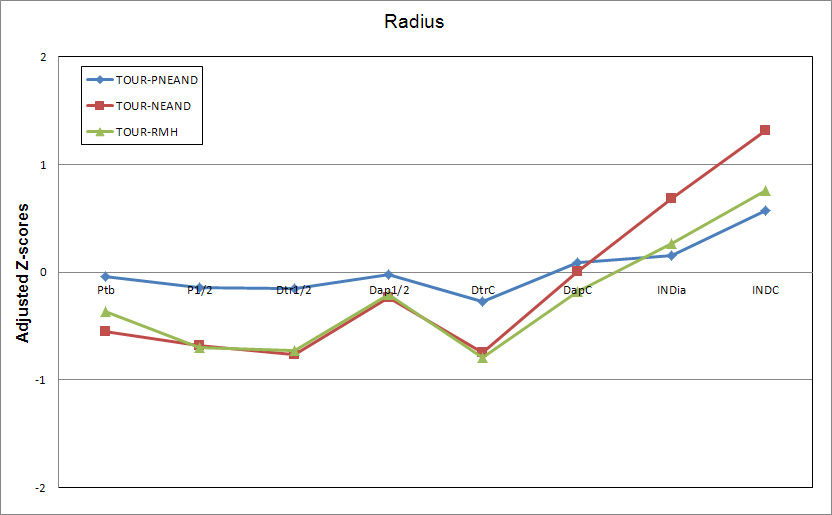


Figure S11.
