# Supplementary figures and images for: Middle Pleistocene Human Remains from Tourville-la-Rivière (Normandy, France) and Their Archaeological Context
Source: PLoS One. 2014 Oct 8;9(10):e104111. doi: 10.1371/journal.pone.0104111 (PMC4189787; doi:10.1371/journal.pone.0104111)

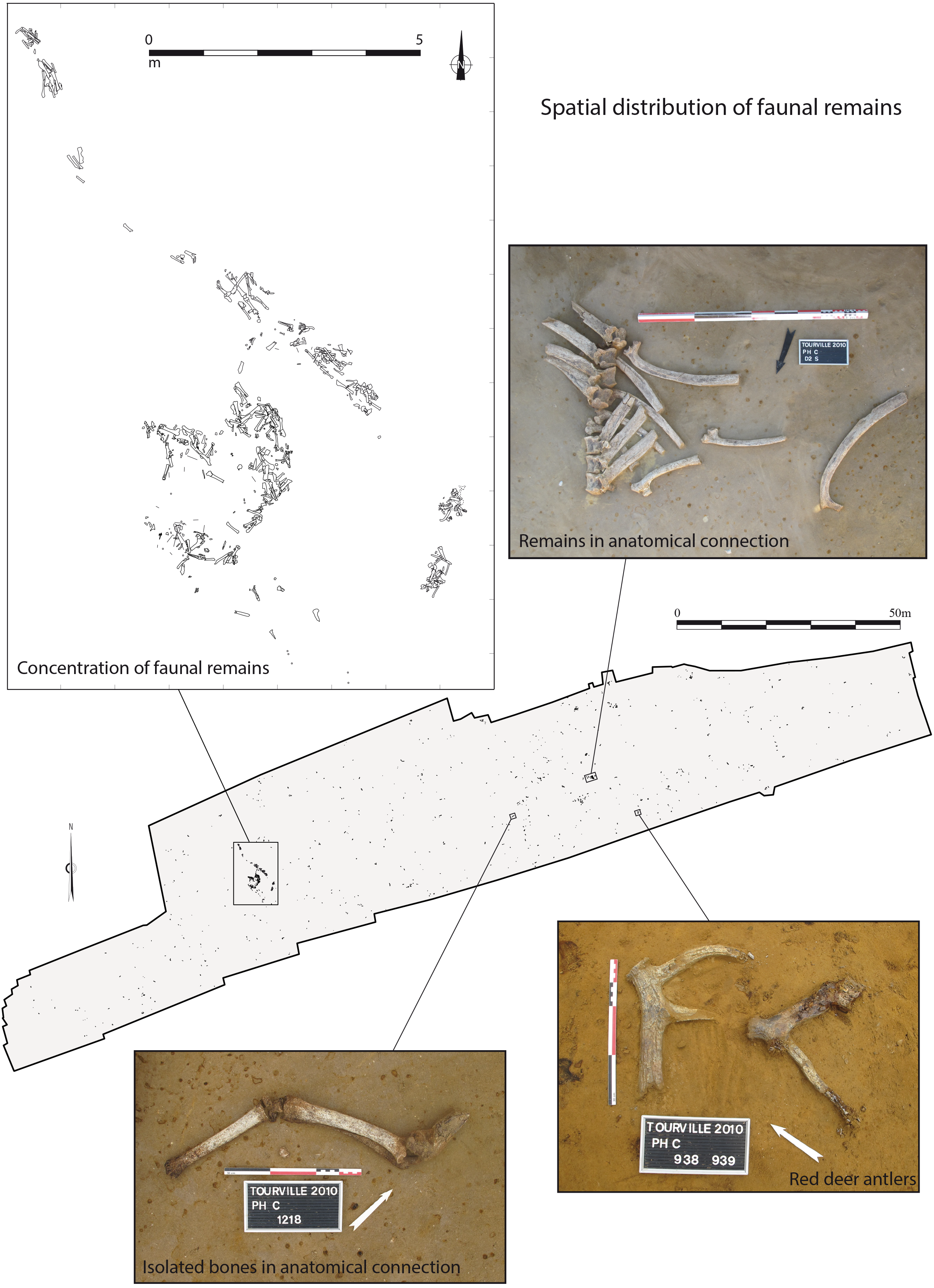

Supplement: File S1 — Headings and captions of the supporting text, supporting figures, and supporting tables. Text S1. Surface alteration of the lithic artefacts. Text S2. The Tourville example of non-Levallois laminar debitage. Text S3. Preliminary use-wear results. Text S4. U-series and ESR analyses. Text S5. Preservation of the Tourville fossils. Text S6. Comparison groups used in the morphometric analysis. Text S7. CT-scan methodology and results. Figure S1.Spatial distribution of the faunal remains. Figure S2. The D2 inf faunal assemblage. Figure S3. Spatial distribution of lithic artefacts and focus on the knapping area. Figure S4. Refitting sequence comprising 46 pieces from the knapping concentration (a). While most elements of the reduction sequence are represented (waste, core management and shaping flakes, fragments of flakes and blades), several refitting sequences (b and c) show that the cores and largest products were exported. Figure S5. Rocourt-type debitage. 1- Elongated éclats débordants refit with laminar flake fragments. The negatives evince a bipolar debitage method producing either laminar flakes or blades. 2, 3 – Rocourt-type blades. Figure S6. Examples of macro-wear (scarring) on Levallois products probably used to work soft animal materials. Figure S7.1. U-series results of five bone fragments of the human remains. Top left: sample holder before analysis, left column: laser ablation analysis spots (the spot diameters are around 250 µm across); right column: U-series isotope results. When the 230Th/238U ratio is >234U/238U then leaching has occurred and no age can be calculated. Figure S7.2. U-series results on eight faunal teeth. Left: photos on the cross sections with laser ablation pits. The arrows indicate the analysis direction. Middle column: U-series isotope results. Right column: apparent U-series age estimates. Leaching is indicated by 400 ka age estimates, U-concentrations too low for age calculation are shown as zero ages. Figure S8. Schematic represen [file pone.0104111.s001.zip › Figures SI High Resolution/Figure S1.tif]

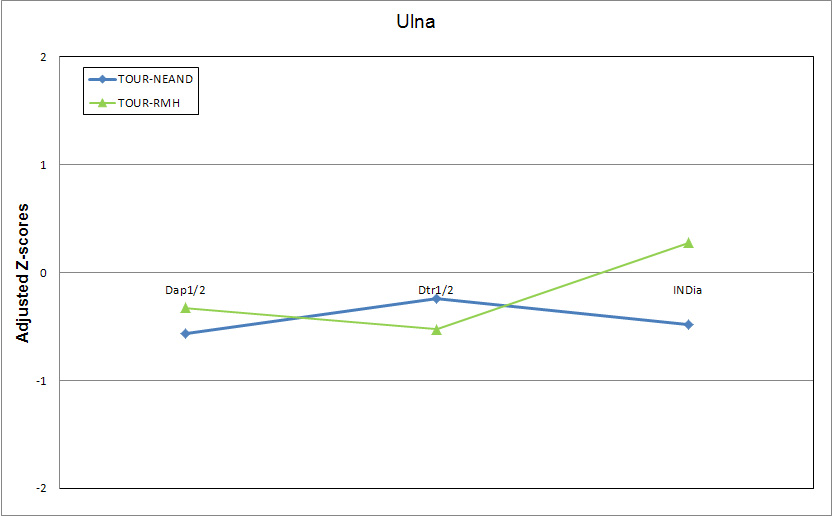

Supplement: File S1 — Headings and captions of the supporting text, supporting figures, and supporting tables. Text S1. Surface alteration of the lithic artefacts. Text S2. The Tourville example of non-Levallois laminar debitage. Text S3. Preliminary use-wear results. Text S4. U-series and ESR analyses. Text S5. Preservation of the Tourville fossils. Text S6. Comparison groups used in the morphometric analysis. Text S7. CT-scan methodology and results. Figure S1.Spatial distribution of the faunal remains. Figure S2. The D2 inf faunal assemblage. Figure S3. Spatial distribution of lithic artefacts and focus on the knapping area. Figure S4. Refitting sequence comprising 46 pieces from the knapping concentration (a). While most elements of the reduction sequence are represented (waste, core management and shaping flakes, fragments of flakes and blades), several refitting sequences (b and c) show that the cores and largest products were exported. Figure S5. Rocourt-type debitage. 1- Elongated éclats débordants refit with laminar flake fragments. The negatives evince a bipolar debitage method producing either laminar flakes or blades. 2, 3 – Rocourt-type blades. Figure S6. Examples of macro-wear (scarring) on Levallois products probably used to work soft animal materials. Figure S7.1. U-series results of five bone fragments of the human remains. Top left: sample holder before analysis, left column: laser ablation analysis spots (the spot diameters are around 250 µm across); right column: U-series isotope results. When the 230Th/238U ratio is >234U/238U then leaching has occurred and no age can be calculated. Figure S7.2. U-series results on eight faunal teeth. Left: photos on the cross sections with laser ablation pits. The arrows indicate the analysis direction. Middle column: U-series isotope results. Right column: apparent U-series age estimates. Leaching is indicated by 400 ka age estimates, U-concentrations too low for age calculation are shown as zero ages. Figure S8. Schematic represen [file pone.0104111.s001.zip › Figures SI High Resolution/Figure S10.tif]

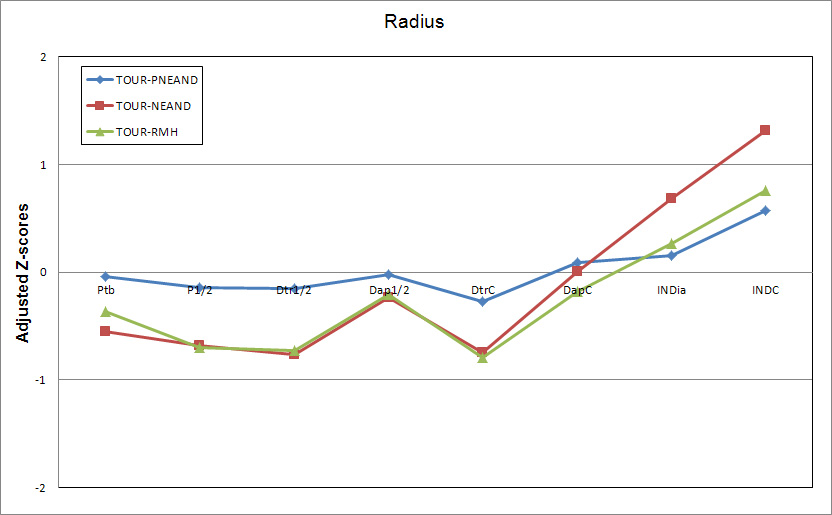

Supplement: File S1 — Headings and captions of the supporting text, supporting figures, and supporting tables. Text S1. Surface alteration of the lithic artefacts. Text S2. The Tourville example of non-Levallois laminar debitage. Text S3. Preliminary use-wear results. Text S4. U-series and ESR analyses. Text S5. Preservation of the Tourville fossils. Text S6. Comparison groups used in the morphometric analysis. Text S7. CT-scan methodology and results. Figure S1.Spatial distribution of the faunal remains. Figure S2. The D2 inf faunal assemblage. Figure S3. Spatial distribution of lithic artefacts and focus on the knapping area. Figure S4. Refitting sequence comprising 46 pieces from the knapping concentration (a). While most elements of the reduction sequence are represented (waste, core management and shaping flakes, fragments of flakes and blades), several refitting sequences (b and c) show that the cores and largest products were exported. Figure S5. Rocourt-type debitage. 1- Elongated éclats débordants refit with laminar flake fragments. The negatives evince a bipolar debitage method producing either laminar flakes or blades. 2, 3 – Rocourt-type blades. Figure S6. Examples of macro-wear (scarring) on Levallois products probably used to work soft animal materials. Figure S7.1. U-series results of five bone fragments of the human remains. Top left: sample holder before analysis, left column: laser ablation analysis spots (the spot diameters are around 250 µm across); right column: U-series isotope results. When the 230Th/238U ratio is >234U/238U then leaching has occurred and no age can be calculated. Figure S7.2. U-series results on eight faunal teeth. Left: photos on the cross sections with laser ablation pits. The arrows indicate the analysis direction. Middle column: U-series isotope results. Right column: apparent U-series age estimates. Leaching is indicated by 400 ka age estimates, U-concentrations too low for age calculation are shown as zero ages. Figure S8. Schematic represen [file pone.0104111.s001.zip › Figures SI High Resolution/Figure S11.tif]

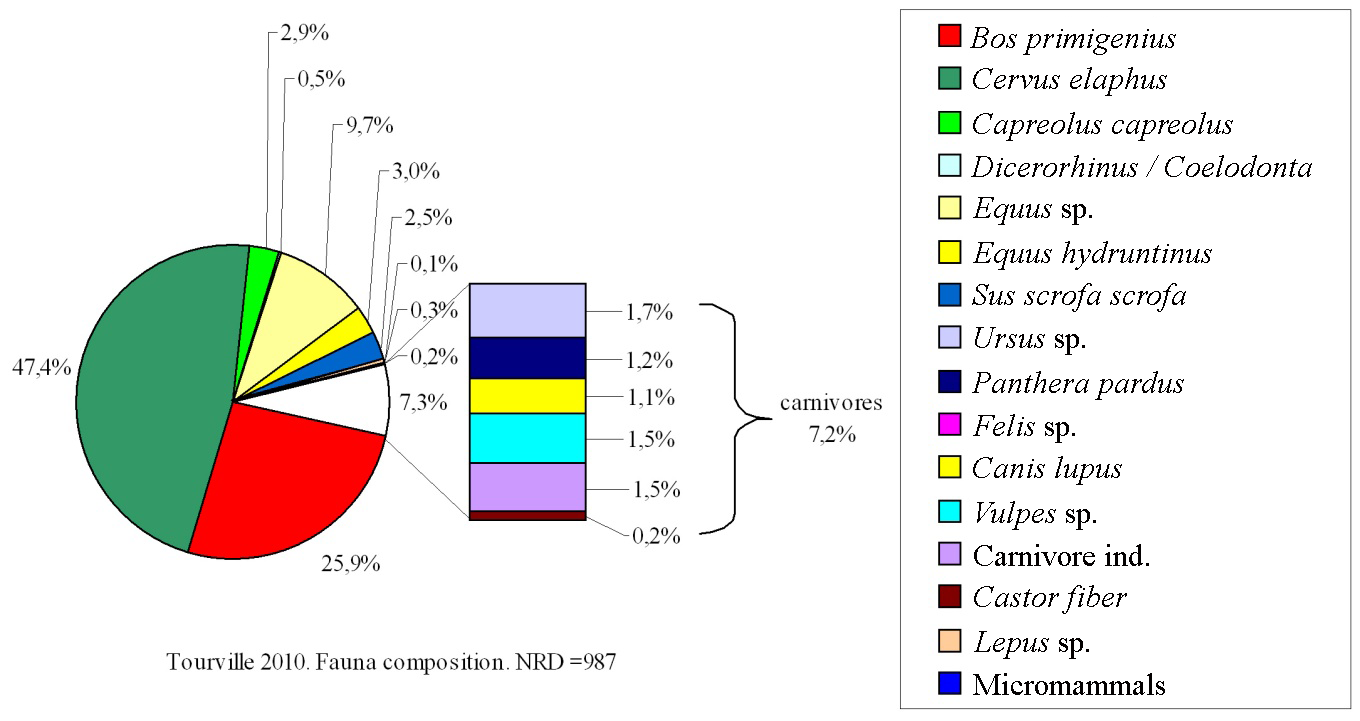

Supplement: File S1 — Headings and captions of the supporting text, supporting figures, and supporting tables. Text S1. Surface alteration of the lithic artefacts. Text S2. The Tourville example of non-Levallois laminar debitage. Text S3. Preliminary use-wear results. Text S4. U-series and ESR analyses. Text S5. Preservation of the Tourville fossils. Text S6. Comparison groups used in the morphometric analysis. Text S7. CT-scan methodology and results. Figure S1.Spatial distribution of the faunal remains. Figure S2. The D2 inf faunal assemblage. Figure S3. Spatial distribution of lithic artefacts and focus on the knapping area. Figure S4. Refitting sequence comprising 46 pieces from the knapping concentration (a). While most elements of the reduction sequence are represented (waste, core management and shaping flakes, fragments of flakes and blades), several refitting sequences (b and c) show that the cores and largest products were exported. Figure S5. Rocourt-type debitage. 1- Elongated éclats débordants refit with laminar flake fragments. The negatives evince a bipolar debitage method producing either laminar flakes or blades. 2, 3 – Rocourt-type blades. Figure S6. Examples of macro-wear (scarring) on Levallois products probably used to work soft animal materials. Figure S7.1. U-series results of five bone fragments of the human remains. Top left: sample holder before analysis, left column: laser ablation analysis spots (the spot diameters are around 250 µm across); right column: U-series isotope results. When the 230Th/238U ratio is >234U/238U then leaching has occurred and no age can be calculated. Figure S7.2. U-series results on eight faunal teeth. Left: photos on the cross sections with laser ablation pits. The arrows indicate the analysis direction. Middle column: U-series isotope results. Right column: apparent U-series age estimates. Leaching is indicated by 400 ka age estimates, U-concentrations too low for age calculation are shown as zero ages. Figure S8. Schematic represen [file pone.0104111.s001.zip › Figures SI High Resolution/Figure S2.tif]

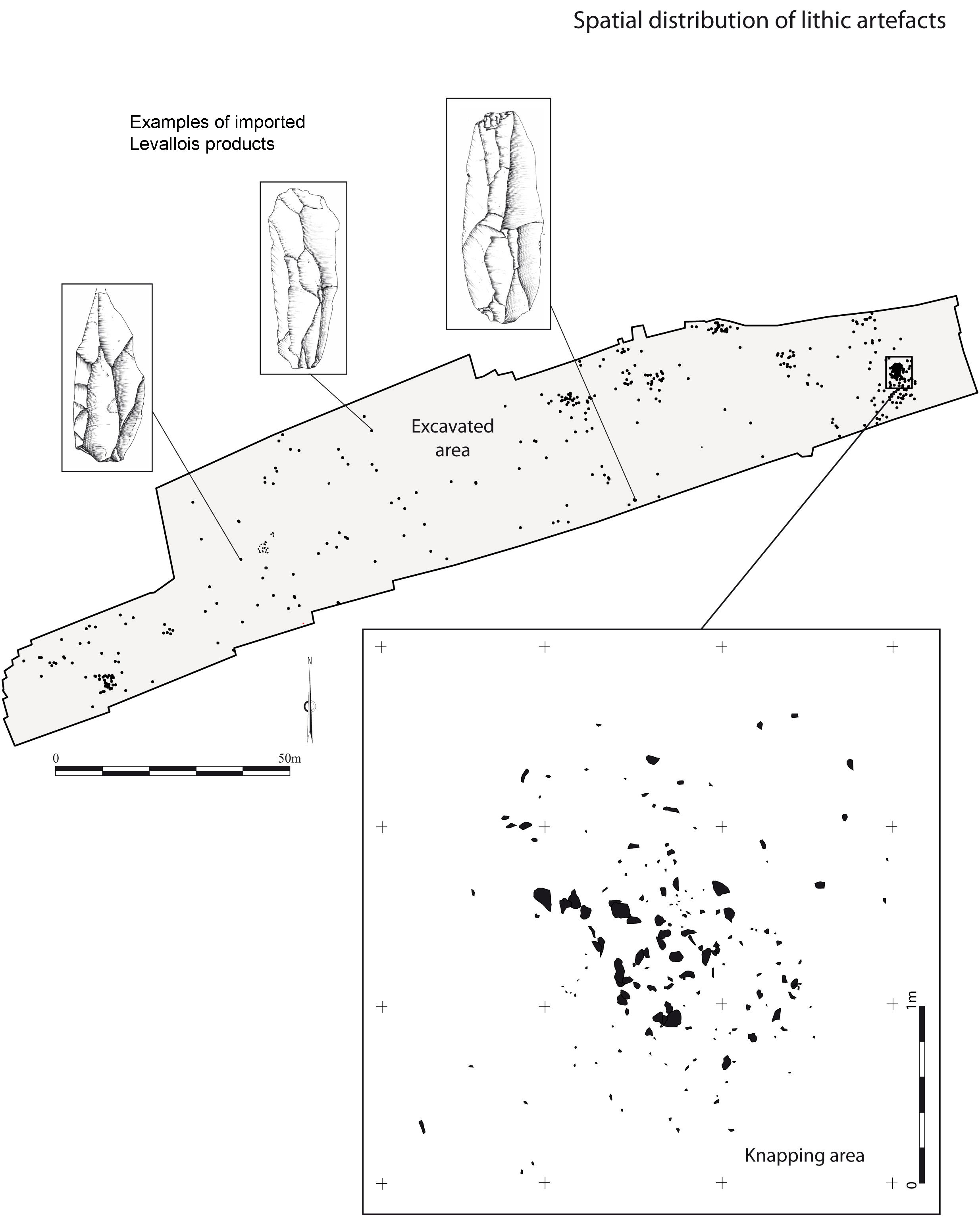

Supplement: File S1 — Headings and captions of the supporting text, supporting figures, and supporting tables. Text S1. Surface alteration of the lithic artefacts. Text S2. The Tourville example of non-Levallois laminar debitage. Text S3. Preliminary use-wear results. Text S4. U-series and ESR analyses. Text S5. Preservation of the Tourville fossils. Text S6. Comparison groups used in the morphometric analysis. Text S7. CT-scan methodology and results. Figure S1.Spatial distribution of the faunal remains. Figure S2. The D2 inf faunal assemblage. Figure S3. Spatial distribution of lithic artefacts and focus on the knapping area. Figure S4. Refitting sequence comprising 46 pieces from the knapping concentration (a). While most elements of the reduction sequence are represented (waste, core management and shaping flakes, fragments of flakes and blades), several refitting sequences (b and c) show that the cores and largest products were exported. Figure S5. Rocourt-type debitage. 1- Elongated éclats débordants refit with laminar flake fragments. The negatives evince a bipolar debitage method producing either laminar flakes or blades. 2, 3 – Rocourt-type blades. Figure S6. Examples of macro-wear (scarring) on Levallois products probably used to work soft animal materials. Figure S7.1. U-series results of five bone fragments of the human remains. Top left: sample holder before analysis, left column: laser ablation analysis spots (the spot diameters are around 250 µm across); right column: U-series isotope results. When the 230Th/238U ratio is >234U/238U then leaching has occurred and no age can be calculated. Figure S7.2. U-series results on eight faunal teeth. Left: photos on the cross sections with laser ablation pits. The arrows indicate the analysis direction. Middle column: U-series isotope results. Right column: apparent U-series age estimates. Leaching is indicated by 400 ka age estimates, U-concentrations too low for age calculation are shown as zero ages. Figure S8. Schematic represen [file pone.0104111.s001.zip › Figures SI High Resolution/Figure S3.tif]

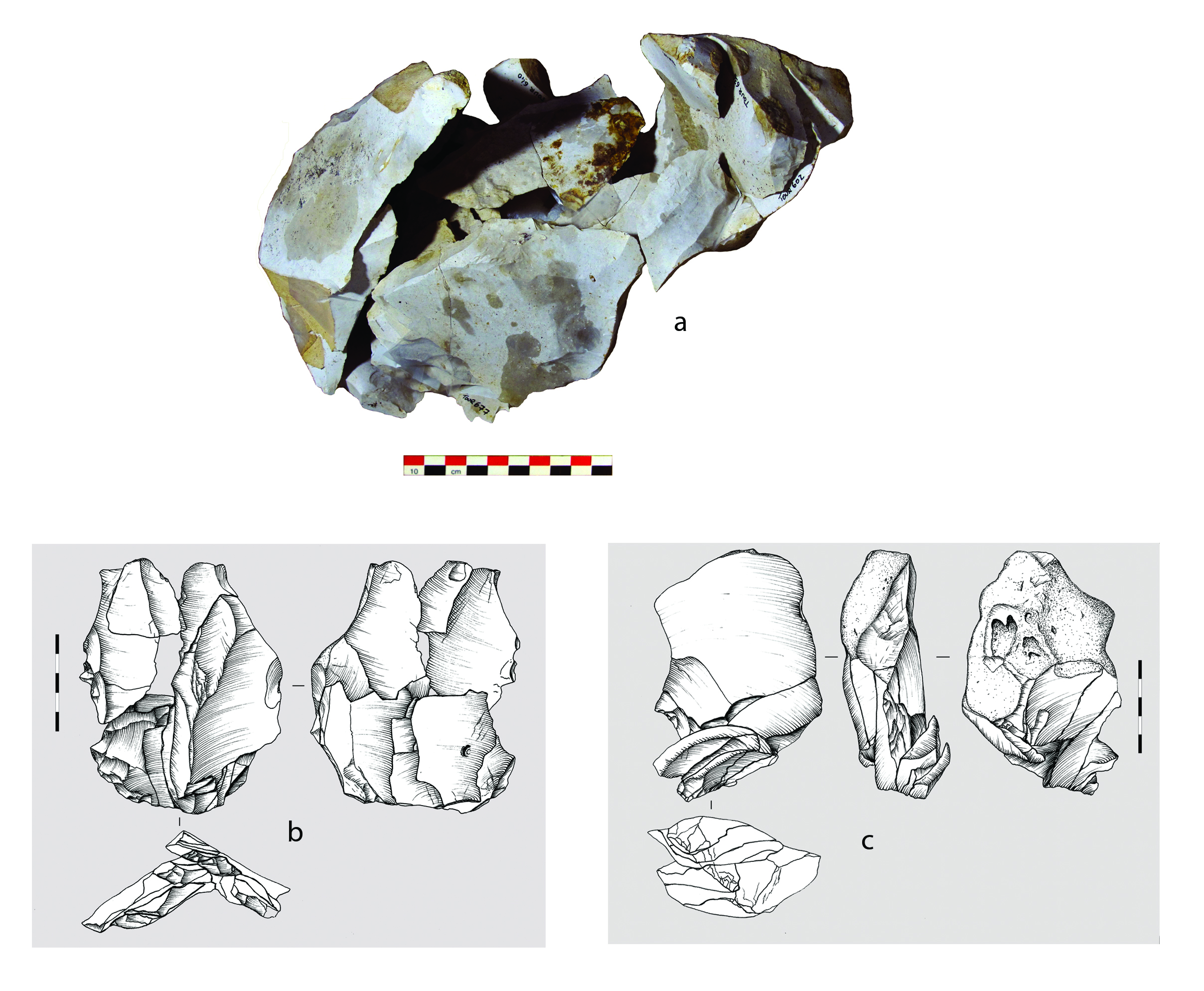

Supplement: File S1 — Headings and captions of the supporting text, supporting figures, and supporting tables. Text S1. Surface alteration of the lithic artefacts. Text S2. The Tourville example of non-Levallois laminar debitage. Text S3. Preliminary use-wear results. Text S4. U-series and ESR analyses. Text S5. Preservation of the Tourville fossils. Text S6. Comparison groups used in the morphometric analysis. Text S7. CT-scan methodology and results. Figure S1.Spatial distribution of the faunal remains. Figure S2. The D2 inf faunal assemblage. Figure S3. Spatial distribution of lithic artefacts and focus on the knapping area. Figure S4. Refitting sequence comprising 46 pieces from the knapping concentration (a). While most elements of the reduction sequence are represented (waste, core management and shaping flakes, fragments of flakes and blades), several refitting sequences (b and c) show that the cores and largest products were exported. Figure S5. Rocourt-type debitage. 1- Elongated éclats débordants refit with laminar flake fragments. The negatives evince a bipolar debitage method producing either laminar flakes or blades. 2, 3 – Rocourt-type blades. Figure S6. Examples of macro-wear (scarring) on Levallois products probably used to work soft animal materials. Figure S7.1. U-series results of five bone fragments of the human remains. Top left: sample holder before analysis, left column: laser ablation analysis spots (the spot diameters are around 250 µm across); right column: U-series isotope results. When the 230Th/238U ratio is >234U/238U then leaching has occurred and no age can be calculated. Figure S7.2. U-series results on eight faunal teeth. Left: photos on the cross sections with laser ablation pits. The arrows indicate the analysis direction. Middle column: U-series isotope results. Right column: apparent U-series age estimates. Leaching is indicated by 400 ka age estimates, U-concentrations too low for age calculation are shown as zero ages. Figure S8. Schematic represen [file pone.0104111.s001.zip › Figures SI High Resolution/Figure S4.tif]

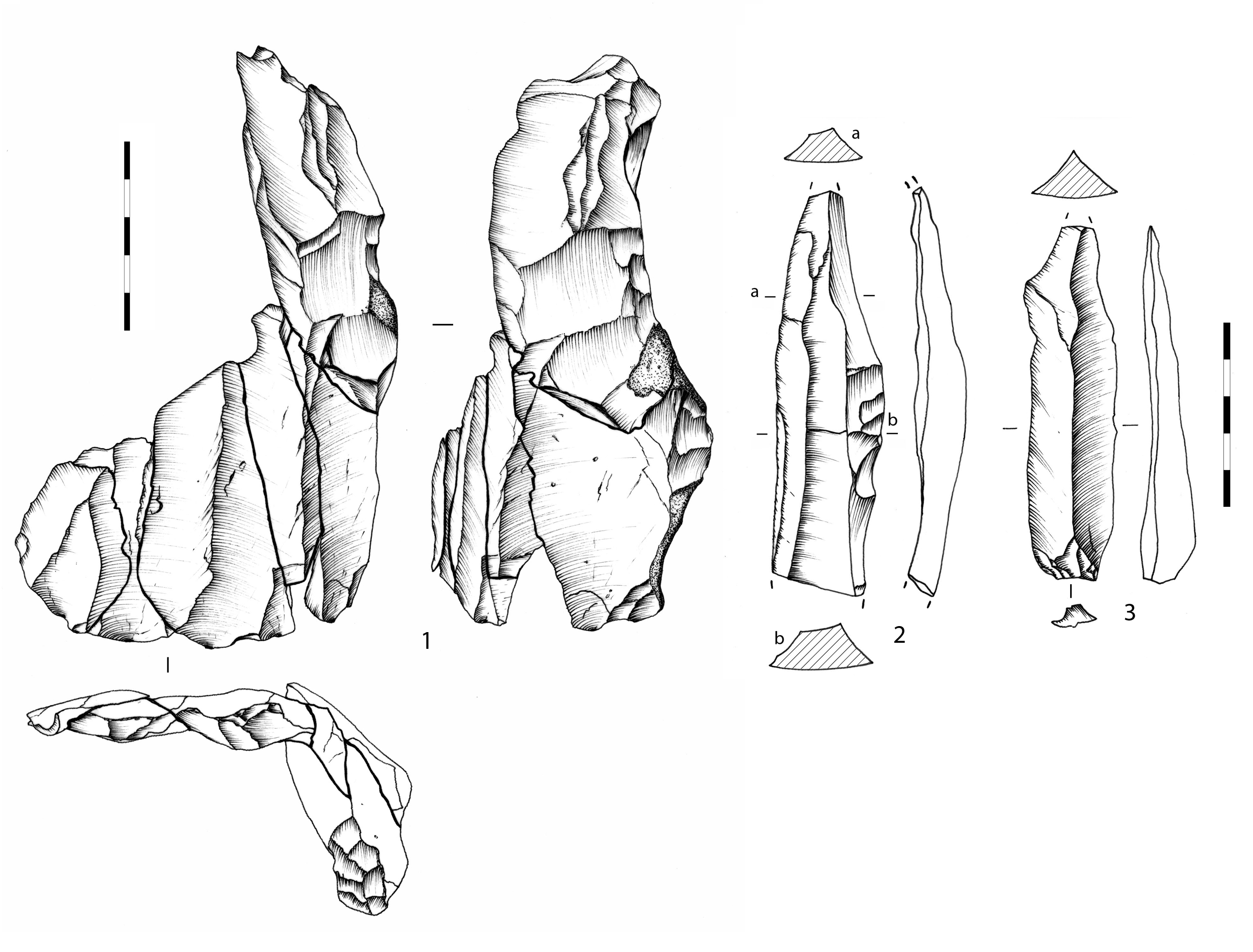

Supplement: File S1 — Headings and captions of the supporting text, supporting figures, and supporting tables. Text S1. Surface alteration of the lithic artefacts. Text S2. The Tourville example of non-Levallois laminar debitage. Text S3. Preliminary use-wear results. Text S4. U-series and ESR analyses. Text S5. Preservation of the Tourville fossils. Text S6. Comparison groups used in the morphometric analysis. Text S7. CT-scan methodology and results. Figure S1.Spatial distribution of the faunal remains. Figure S2. The D2 inf faunal assemblage. Figure S3. Spatial distribution of lithic artefacts and focus on the knapping area. Figure S4. Refitting sequence comprising 46 pieces from the knapping concentration (a). While most elements of the reduction sequence are represented (waste, core management and shaping flakes, fragments of flakes and blades), several refitting sequences (b and c) show that the cores and largest products were exported. Figure S5. Rocourt-type debitage. 1- Elongated éclats débordants refit with laminar flake fragments. The negatives evince a bipolar debitage method producing either laminar flakes or blades. 2, 3 – Rocourt-type blades. Figure S6. Examples of macro-wear (scarring) on Levallois products probably used to work soft animal materials. Figure S7.1. U-series results of five bone fragments of the human remains. Top left: sample holder before analysis, left column: laser ablation analysis spots (the spot diameters are around 250 µm across); right column: U-series isotope results. When the 230Th/238U ratio is >234U/238U then leaching has occurred and no age can be calculated. Figure S7.2. U-series results on eight faunal teeth. Left: photos on the cross sections with laser ablation pits. The arrows indicate the analysis direction. Middle column: U-series isotope results. Right column: apparent U-series age estimates. Leaching is indicated by 400 ka age estimates, U-concentrations too low for age calculation are shown as zero ages. Figure S8. Schematic represen [file pone.0104111.s001.zip › Figures SI High Resolution/Figure S5.tif]

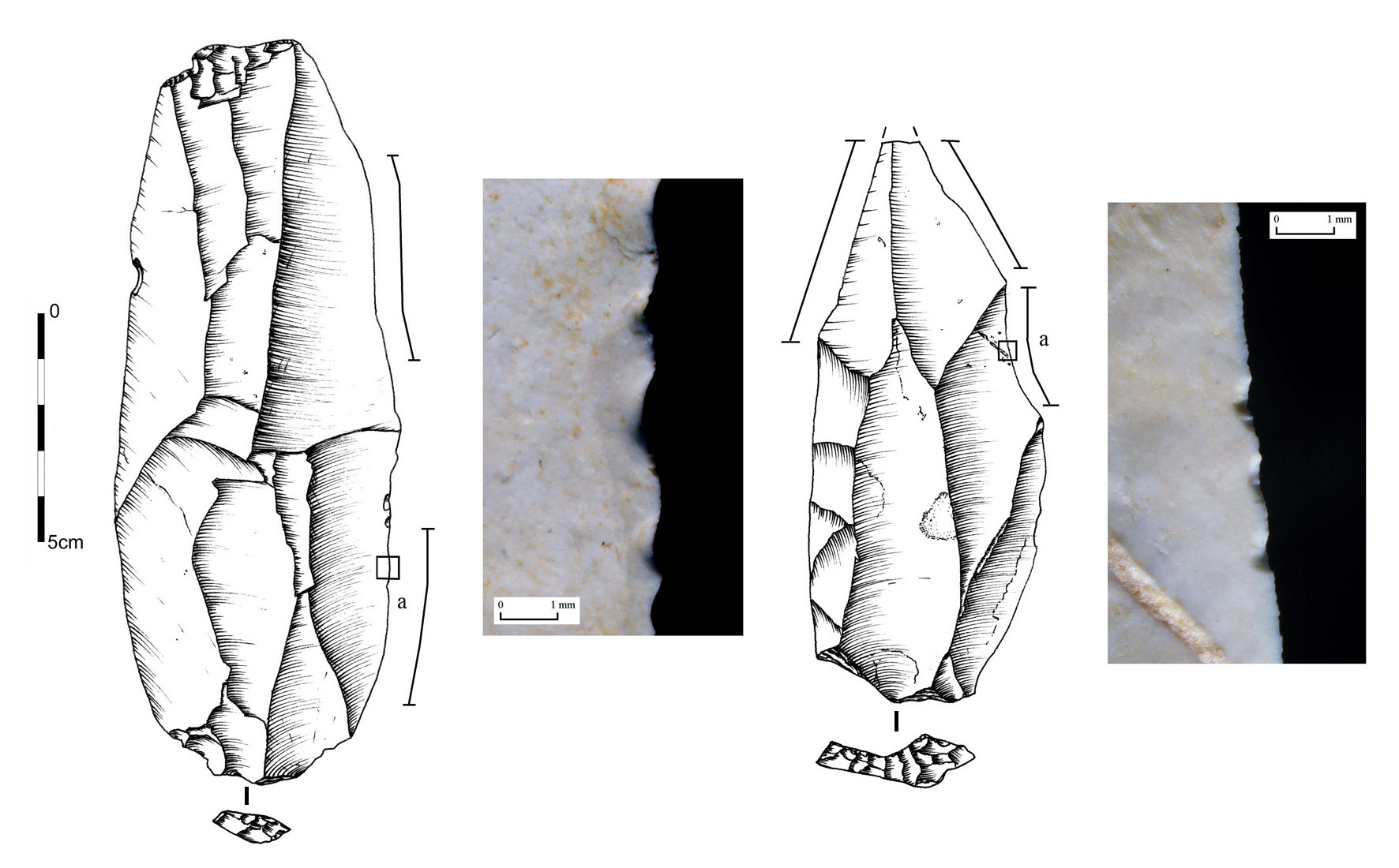

Supplement: File S1 — Headings and captions of the supporting text, supporting figures, and supporting tables. Text S1. Surface alteration of the lithic artefacts. Text S2. The Tourville example of non-Levallois laminar debitage. Text S3. Preliminary use-wear results. Text S4. U-series and ESR analyses. Text S5. Preservation of the Tourville fossils. Text S6. Comparison groups used in the morphometric analysis. Text S7. CT-scan methodology and results. Figure S1.Spatial distribution of the faunal remains. Figure S2. The D2 inf faunal assemblage. Figure S3. Spatial distribution of lithic artefacts and focus on the knapping area. Figure S4. Refitting sequence comprising 46 pieces from the knapping concentration (a). While most elements of the reduction sequence are represented (waste, core management and shaping flakes, fragments of flakes and blades), several refitting sequences (b and c) show that the cores and largest products were exported. Figure S5. Rocourt-type debitage. 1- Elongated éclats débordants refit with laminar flake fragments. The negatives evince a bipolar debitage method producing either laminar flakes or blades. 2, 3 – Rocourt-type blades. Figure S6. Examples of macro-wear (scarring) on Levallois products probably used to work soft animal materials. Figure S7.1. U-series results of five bone fragments of the human remains. Top left: sample holder before analysis, left column: laser ablation analysis spots (the spot diameters are around 250 µm across); right column: U-series isotope results. When the 230Th/238U ratio is >234U/238U then leaching has occurred and no age can be calculated. Figure S7.2. U-series results on eight faunal teeth. Left: photos on the cross sections with laser ablation pits. The arrows indicate the analysis direction. Middle column: U-series isotope results. Right column: apparent U-series age estimates. Leaching is indicated by 400 ka age estimates, U-concentrations too low for age calculation are shown as zero ages. Figure S8. Schematic represen [file pone.0104111.s001.zip › Figures SI High Resolution/Figure S6.tif]

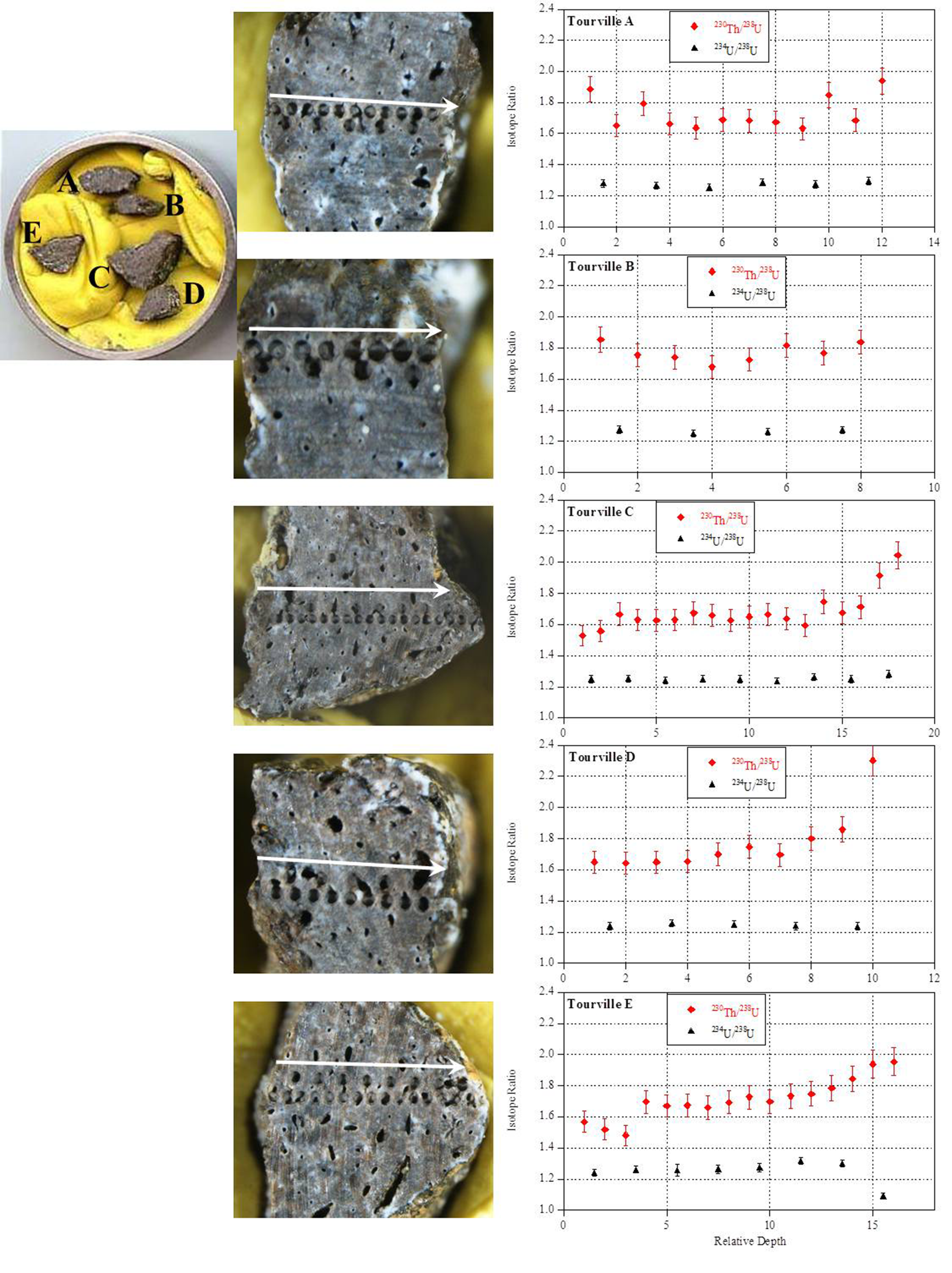

Supplement: File S1 — Headings and captions of the supporting text, supporting figures, and supporting tables. Text S1. Surface alteration of the lithic artefacts. Text S2. The Tourville example of non-Levallois laminar debitage. Text S3. Preliminary use-wear results. Text S4. U-series and ESR analyses. Text S5. Preservation of the Tourville fossils. Text S6. Comparison groups used in the morphometric analysis. Text S7. CT-scan methodology and results. Figure S1.Spatial distribution of the faunal remains. Figure S2. The D2 inf faunal assemblage. Figure S3. Spatial distribution of lithic artefacts and focus on the knapping area. Figure S4. Refitting sequence comprising 46 pieces from the knapping concentration (a). While most elements of the reduction sequence are represented (waste, core management and shaping flakes, fragments of flakes and blades), several refitting sequences (b and c) show that the cores and largest products were exported. Figure S5. Rocourt-type debitage. 1- Elongated éclats débordants refit with laminar flake fragments. The negatives evince a bipolar debitage method producing either laminar flakes or blades. 2, 3 – Rocourt-type blades. Figure S6. Examples of macro-wear (scarring) on Levallois products probably used to work soft animal materials. Figure S7.1. U-series results of five bone fragments of the human remains. Top left: sample holder before analysis, left column: laser ablation analysis spots (the spot diameters are around 250 µm across); right column: U-series isotope results. When the 230Th/238U ratio is >234U/238U then leaching has occurred and no age can be calculated. Figure S7.2. U-series results on eight faunal teeth. Left: photos on the cross sections with laser ablation pits. The arrows indicate the analysis direction. Middle column: U-series isotope results. Right column: apparent U-series age estimates. Leaching is indicated by 400 ka age estimates, U-concentrations too low for age calculation are shown as zero ages. Figure S8. Schematic represen [file pone.0104111.s001.zip › Figures SI High Resolution/Figure S7_1.tif]

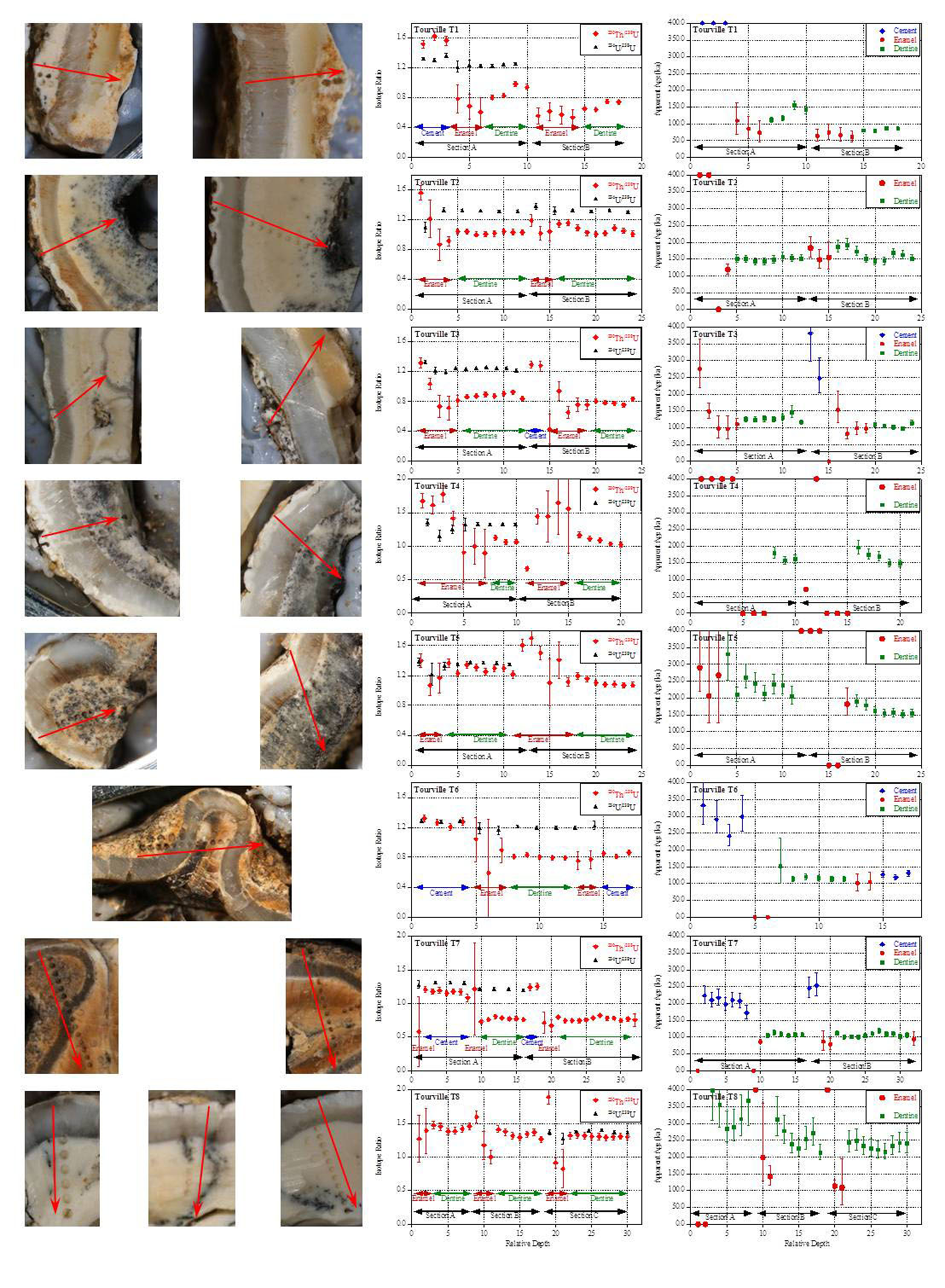

Supplement: File S1 — Headings and captions of the supporting text, supporting figures, and supporting tables. Text S1. Surface alteration of the lithic artefacts. Text S2. The Tourville example of non-Levallois laminar debitage. Text S3. Preliminary use-wear results. Text S4. U-series and ESR analyses. Text S5. Preservation of the Tourville fossils. Text S6. Comparison groups used in the morphometric analysis. Text S7. CT-scan methodology and results. Figure S1.Spatial distribution of the faunal remains. Figure S2. The D2 inf faunal assemblage. Figure S3. Spatial distribution of lithic artefacts and focus on the knapping area. Figure S4. Refitting sequence comprising 46 pieces from the knapping concentration (a). While most elements of the reduction sequence are represented (waste, core management and shaping flakes, fragments of flakes and blades), several refitting sequences (b and c) show that the cores and largest products were exported. Figure S5. Rocourt-type debitage. 1- Elongated éclats débordants refit with laminar flake fragments. The negatives evince a bipolar debitage method producing either laminar flakes or blades. 2, 3 – Rocourt-type blades. Figure S6. Examples of macro-wear (scarring) on Levallois products probably used to work soft animal materials. Figure S7.1. U-series results of five bone fragments of the human remains. Top left: sample holder before analysis, left column: laser ablation analysis spots (the spot diameters are around 250 µm across); right column: U-series isotope results. When the 230Th/238U ratio is >234U/238U then leaching has occurred and no age can be calculated. Figure S7.2. U-series results on eight faunal teeth. Left: photos on the cross sections with laser ablation pits. The arrows indicate the analysis direction. Middle column: U-series isotope results. Right column: apparent U-series age estimates. Leaching is indicated by 400 ka age estimates, U-concentrations too low for age calculation are shown as zero ages. Figure S8. Schematic represen [file pone.0104111.s001.zip › Figures SI High Resolution/Figure S7_2.tif]

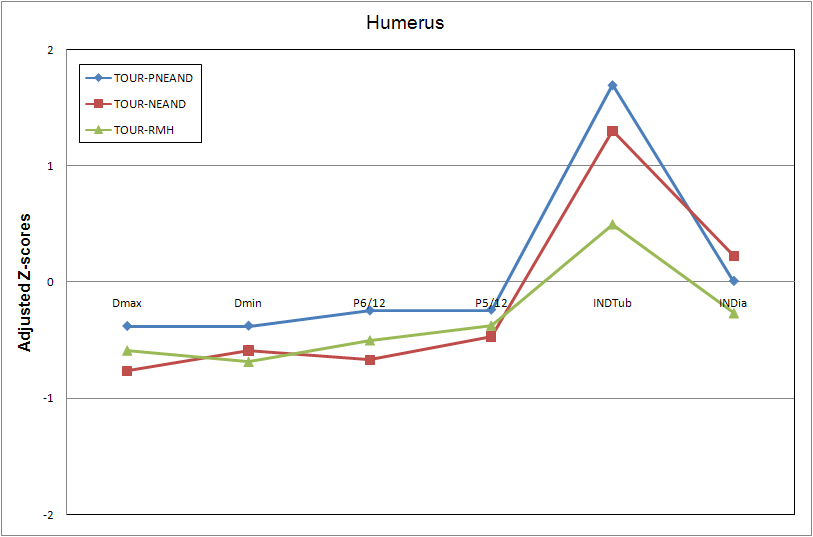

Supplement: File S1 — Headings and captions of the supporting text, supporting figures, and supporting tables. Text S1. Surface alteration of the lithic artefacts. Text S2. The Tourville example of non-Levallois laminar debitage. Text S3. Preliminary use-wear results. Text S4. U-series and ESR analyses. Text S5. Preservation of the Tourville fossils. Text S6. Comparison groups used in the morphometric analysis. Text S7. CT-scan methodology and results. Figure S1.Spatial distribution of the faunal remains. Figure S2. The D2 inf faunal assemblage. Figure S3. Spatial distribution of lithic artefacts and focus on the knapping area. Figure S4. Refitting sequence comprising 46 pieces from the knapping concentration (a). While most elements of the reduction sequence are represented (waste, core management and shaping flakes, fragments of flakes and blades), several refitting sequences (b and c) show that the cores and largest products were exported. Figure S5. Rocourt-type debitage. 1- Elongated éclats débordants refit with laminar flake fragments. The negatives evince a bipolar debitage method producing either laminar flakes or blades. 2, 3 – Rocourt-type blades. Figure S6. Examples of macro-wear (scarring) on Levallois products probably used to work soft animal materials. Figure S7.1. U-series results of five bone fragments of the human remains. Top left: sample holder before analysis, left column: laser ablation analysis spots (the spot diameters are around 250 µm across); right column: U-series isotope results. When the 230Th/238U ratio is >234U/238U then leaching has occurred and no age can be calculated. Figure S7.2. U-series results on eight faunal teeth. Left: photos on the cross sections with laser ablation pits. The arrows indicate the analysis direction. Middle column: U-series isotope results. Right column: apparent U-series age estimates. Leaching is indicated by 400 ka age estimates, U-concentrations too low for age calculation are shown as zero ages. Figure S8. Schematic represen [file pone.0104111.s001.zip › Figures SI High Resolution/Figure S8.tif]

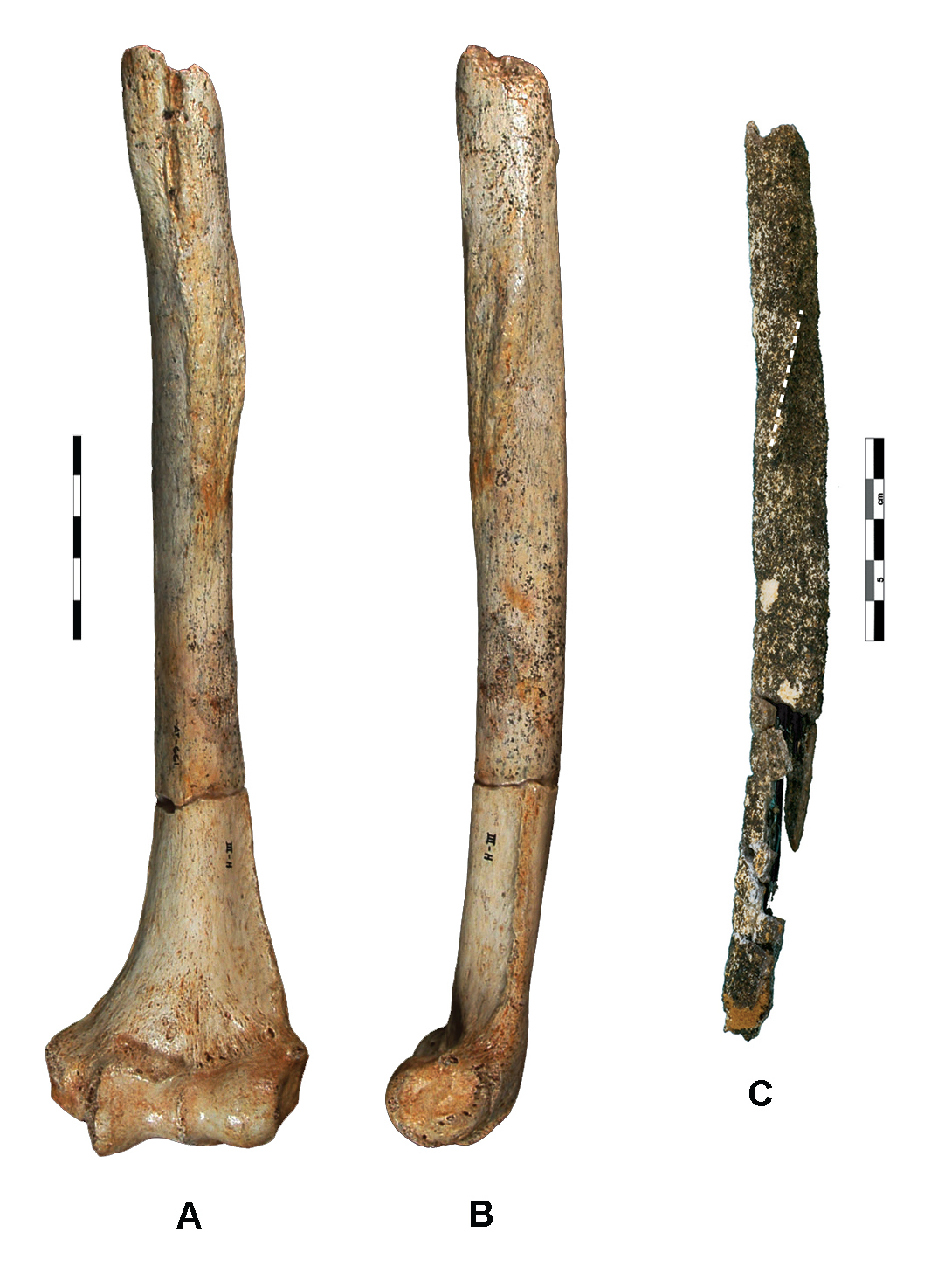

Supplement: File S1 — Headings and captions of the supporting text, supporting figures, and supporting tables. Text S1. Surface alteration of the lithic artefacts. Text S2. The Tourville example of non-Levallois laminar debitage. Text S3. Preliminary use-wear results. Text S4. U-series and ESR analyses. Text S5. Preservation of the Tourville fossils. Text S6. Comparison groups used in the morphometric analysis. Text S7. CT-scan methodology and results. Figure S1.Spatial distribution of the faunal remains. Figure S2. The D2 inf faunal assemblage. Figure S3. Spatial distribution of lithic artefacts and focus on the knapping area. Figure S4. Refitting sequence comprising 46 pieces from the knapping concentration (a). While most elements of the reduction sequence are represented (waste, core management and shaping flakes, fragments of flakes and blades), several refitting sequences (b and c) show that the cores and largest products were exported. Figure S5. Rocourt-type debitage. 1- Elongated éclats débordants refit with laminar flake fragments. The negatives evince a bipolar debitage method producing either laminar flakes or blades. 2, 3 – Rocourt-type blades. Figure S6. Examples of macro-wear (scarring) on Levallois products probably used to work soft animal materials. Figure S7.1. U-series results of five bone fragments of the human remains. Top left: sample holder before analysis, left column: laser ablation analysis spots (the spot diameters are around 250 µm across); right column: U-series isotope results. When the 230Th/238U ratio is >234U/238U then leaching has occurred and no age can be calculated. Figure S7.2. U-series results on eight faunal teeth. Left: photos on the cross sections with laser ablation pits. The arrows indicate the analysis direction. Middle column: U-series isotope results. Right column: apparent U-series age estimates. Leaching is indicated by 400 ka age estimates, U-concentrations too low for age calculation are shown as zero ages. Figure S8. Schematic represen [file pone.0104111.s001.zip › Figures SI High Resolution/Figure S9.tif]
